# Supporting resilience-based coral reef management using broadscale threshold approaches

**DOI:** 10.1038/s41598-025-09531-9

**Published:** 2025-07-16

**Authors:** April J. Burt, Anna Koester, Nancy Bunbury, Philip Haupt, Rowana Walton, Frauke Fleischer-Dogley, Karen M. Chong-Seng

**Affiliations:** 1 Seychelles Islands Foundation, PO Box 853, Victoria, Seychelles; 2 Marine Ecology Department, Faculty of Biology & Chemistry, University of Bremen, Bremen, Germany; 3 Centre for Ecology and Conservation, University of Exeter, Cornwall Campus, Penryn, TR10 9FE UK; 4 Kent and Essex Inshore Fisheries Conservation Authority, Ramsgate, Kent, CT11 9HD UK; 5 ARC Centre of Excellence for Coral Reef Studies, James Cook University, Townsville, QLD 4811 Australia

**Keywords:** Ecological thresholds, Resilience indicators, Coral reef conservation, Coral bleaching, Coral reef recovery, UNESCO world heritage, Ecosystem ecology, Ecosystem ecology, Environmental impact, Marine biology, Environmental health, Marine biology, Environmental sciences

## Abstract

Resilience-Based Management of coral reefs aims to maintain ecosystem function and maximise resilience. This requires identification of resilience indicators and clear ecological reference thresholds for reef managers to maintain or aim for. In the absence of local thresholds, managers can assess reef condition by comparing locally collected indicator data to broadscale thresholds, which account for spatial and temporal variability. This study assesses reef condition at Aldabra Atoll, a remote MPA in the western Indian Ocean, relative to broadscale thresholds for structural complexity, fish biomass, herbivore biomass, juvenile coral density, and trophic-level fish biomass. Results were synthesized into a resilience index, and sites were classified into ‘management strategies’ using a published reef management framework. Resilience scores were then compared to observe coral cover changes following the 2016 bleaching event, tracking recovery through to 2022. Findings showed that seven of the eight assessed seaward reefs at Aldabra displayed the resilience expected of a remote, well-managed marine reserve. The research station and associated human activity appeared to have minimal negative impacts on reef resilience. We recommend expanding the range of broadscale threshold categories and integrating site-specific factors to improve future assessments and management decisions.

**Supplementary Information:**

The online version contains supplementary material available at 10.1038/s41598-025-09531-9.

## Introduction

In the face of climate change and lagging political responses to reducing greenhouse gas emissions, even some of the best protected coral reefs are transforming rapidly^1–5^. As a result, coral reef conservation and management goals have shifted from maintaining intact systems to preserving ecosystem functions^6–8^ and maximising reef resilience. Reef resilience, in this context, refers to a reef’s ability to resist and recover from disturbances^9^. This approach, known as Resilience-Based Management (RBM) is complex and requires clear articulation of management aims (e.g., to maintain or enhance reef resilience towards large scale climatic disturbances and local scale human pressures; to track the effectiveness of reef management activities), identification of indicators to monitor the specific ecosystem functions, and clear resilience indicator reference thresholds for reef managers to maintain or aim for^6,8,10^.

Resilience indicators aim to measure information about important ecosystem processes, function and identity^11–13^. A range of resilience indicators have been identified through analyses of coral reef responses to past disturbance events^14,15^. These indicators include environmental, social, and biological factors and are strongly correlated with a reef’s capacity to recover or maintain function^16^. To effectively apply RBM, managers need to define resilience indicator thresholds at the beginning of the RBM process as well as considering how to convert results into evidence-based management decision-making (prior to undertaking a resilience potential assessment). However, determining appropriate thresholds depends on access to historical, site-specific data about local marine communities and their natural temporal variability^17,18^ and/or extensive spatial surveys to document the area’s spatial variability^19,20^. The latter method necessarily results in relational threshold values specific to the study area at a single point in time rather than considering historical baselines^11,13^. Unfortunately, historical data are often lacking at the necessary spatial scales, and many management authorities have limited capacity or resources to generate localized thresholds^21,22^.

To counter these challenges, some studies have established broadscale thresholds for resilience indicators by incorporating large spatial and, sometimes also, temporal variability. It is feasible for reef managers with reef monitoring capacity to conduct local-scale resilience assessments by collecting data on resilience indicators and comparing them to these published thresholds. Although there are inherent problems in applying general values to a particular reef site, managers can adopt them as temporary ‘thresholds of potential concern’ until more site-specific data can be acquired^23,24^.

This study documents the process used to kickstart RBM at such a site with no recent historical data. Reef resilience indicators used were based primarily on the availability of associated broadscale thresholds in the period of the resilience assessment (ca. 2015), however, there are increasing options available^25,26^. Graham et al.^27^ established thresholds for reef depth, structural complexity, herbivore biomass and juvenile coral density based on data over 17 years from 21 reef sites across the inner islands of Seychelles. These indicators relate to specific ecosystem functions (Table 1). MacNeil et al.^28^ developed a threshold to define minimum resident reef fish biomass in no-take zones using globally distributed fish biomass data from > 800 windward coral reefs, including from the Seychelles, along an exploitation gradient. This latter indicator was used to determine whether there was evidence of fishing pressure on local fish stocks.

**Table 1:** Components of the resilience and future management assessment showing corresponding ecosystem processes and functions.

Approach	Method	Metric & year of data collection	Process of interest^a^	Indicative of^a^	References	Analysis	
Resilience Assessment	Comparison with published recommended reference thresholds	Total fish biomass (1013 kg ha^−1^ of diurnally active, non-cryptic resident reef fish fish > 10 cm of 20 families); data collected in 2015	Productivity	Community functions	MacNeil et al.^28^ (> 800 reefs, incl. Seychelles)	Two-tailed one-sample t-tests and permutation analysis (see Methods S2) against reference thresholds Scoring of t-test and permutation analysis outcomes Scores used in to generate the composite index	
		Herbivore biomass (177 kg ha^−1^); data collected in 2015	Herbivory	Primary production, community functions	Graham et al.^27^ (21 sites in Seychelles, 17 years)		
		Coral juvenile abundance (>6.2 per m^2^); data collected in 2015	Coral recruitment	Reef recovery (gain of primary habitat)			
		Structural complexity (> 3.1); data collected in 2015	Productivity, active reef growth	Habitat availability, benthic community landscape, enhanced coral settlement			
	Comparisons based on trophic pyramids distributions	Trophic levels of diurnally active, non-cryptic, reef-associated fish; data collected in 2015	Productivity	Human impact (cf. unexploited systems)	Graham et al.^29^(253 reefs, incl. Seychelles)	Comparison of trophic pyramids with those of “fished” and “unfished” seascapes as per Graham et al.^29^ and the classic trophic pyramid as per Trebilco et al.^68^	
Management Assessment	Comparison to established threshold values of heat stress	Water temperature (satellite derived^69^)	Physiological stress	Significant thermal stress	Darling et al.^30^(2,584 reefs, incl. Seychelles)	Assessing local DHW value during the 2014–2017 global bleaching event against 4 DHW threshold	Classification of sites into management strategy portfolio of Darling et al.^30^: protect, recover or transform.
	Comparison against the recommended minimum reference value (10%)	Percent cover of competitive and stress-tolerant corals (framework corals); data collected in 2014	Coral calcification	Active reef growth (gain of primary habitat)		T-test and permutation analysis (see Methods S2) against reference threshold	

^a^Also with reference to Brandl et al.’s^7^ eight core ecosystem processes.

Resilience indicators have also been derived from analysing the structure and composition of reef fish communities, which can reveal ecosystem energy flows (e.g. Graham et al.^29^) and life history characteristics (e.g. Darling et al.^30^) that provide insight into reef successional stage. Graham et al.^29^ used data from 253 Indian Ocean reefs, including in the Seychelles, to model fish trophic distributions across varying levels of human disturbance. This study produced trophic-level biomass benchmarks that managers can compare with local fish populations to infer ecological condition. Although this model does not account for environmental gradient effects on trophic structure—a limitation worth considering (see Heenan et al.^25^)—it provides a valuable framework for assessing reef resilience. Darling et al.^30^ analysed coral data from 2,584 Indo-Pacific reefs to examine how 21 ecological, climatic, and social drivers affect coral assemblages. Indicators such as the percent cover of competitive and stress-tolerant corals, prior heat stress exposure, and heat stress intensity were used to evaluate resilience-related processes (e.g., structural complexity, carbonate production, past thermal exposure). Based on these, reefs were allocated to one of three management strategies: (1) Protect: Reefs with limited recent heat stress (DHW < 4 °C-weeks) and coral cover > 10%; (2) Recover: Reefs with coral cover > 10% but exposed to heat stress above 4 °C-weeks during the 2014–2017 global coral bleaching event; and (3) Transform: Reefs with coral cover < 10% and net-negative carbonate budgets, where societies may need to transition away from reef-dependent livelihoods^30^. Among tools developed for RBM under climate change^6,26,31–33^ Darling et al.^30^ stood out for offering meta-analysis-derived thresholds that can be directly applied by managers at the local scale and without long term data.

Although reef resilience assessments are becoming more widespread, few offer a transparent, structured rationale for selecting indicators^13^. Moreover, to our knowledge, there are no published examples of the specific broadscale thresholds discussed above being applied to evaluate site-level resilience or guide management interventions. This study presents an application of these broadscale threshold frameworks for RBM at Aldabra Atoll, part of the Seychelles archipelago in the western Indian Ocean. Using reef survey data collected in 2014 and 2015—prior to the 2016 bleaching event at Aldabra—we: (1) Assessed the relative resilience of Aldabra’s seaward reef sites by analysing their position within the broadscale frameworks described by Graham et al.^27^ and MacNeil et al.^28^ and their placement along known disturbance gradients as outlined by Graham et al.^29^; (2) Synthesized the various resilience indicators and their thresholds into a ‘composite index’ for each site, and; (3) Classified Aldabra’s seaward reefs into one of the management strategies—protect, recover, or transform—according to Darling et al.^30^. Finally, we evaluate how these resilience assessments align with observed live coral cover and recovery trajectories from 2016 to 2022, in the aftermath of one of the most severe and widespread global coral bleaching events on record^4,34^ which peaked in April 2016 at Aldabra.

## Methods

### Study site

**Fig. 1:**
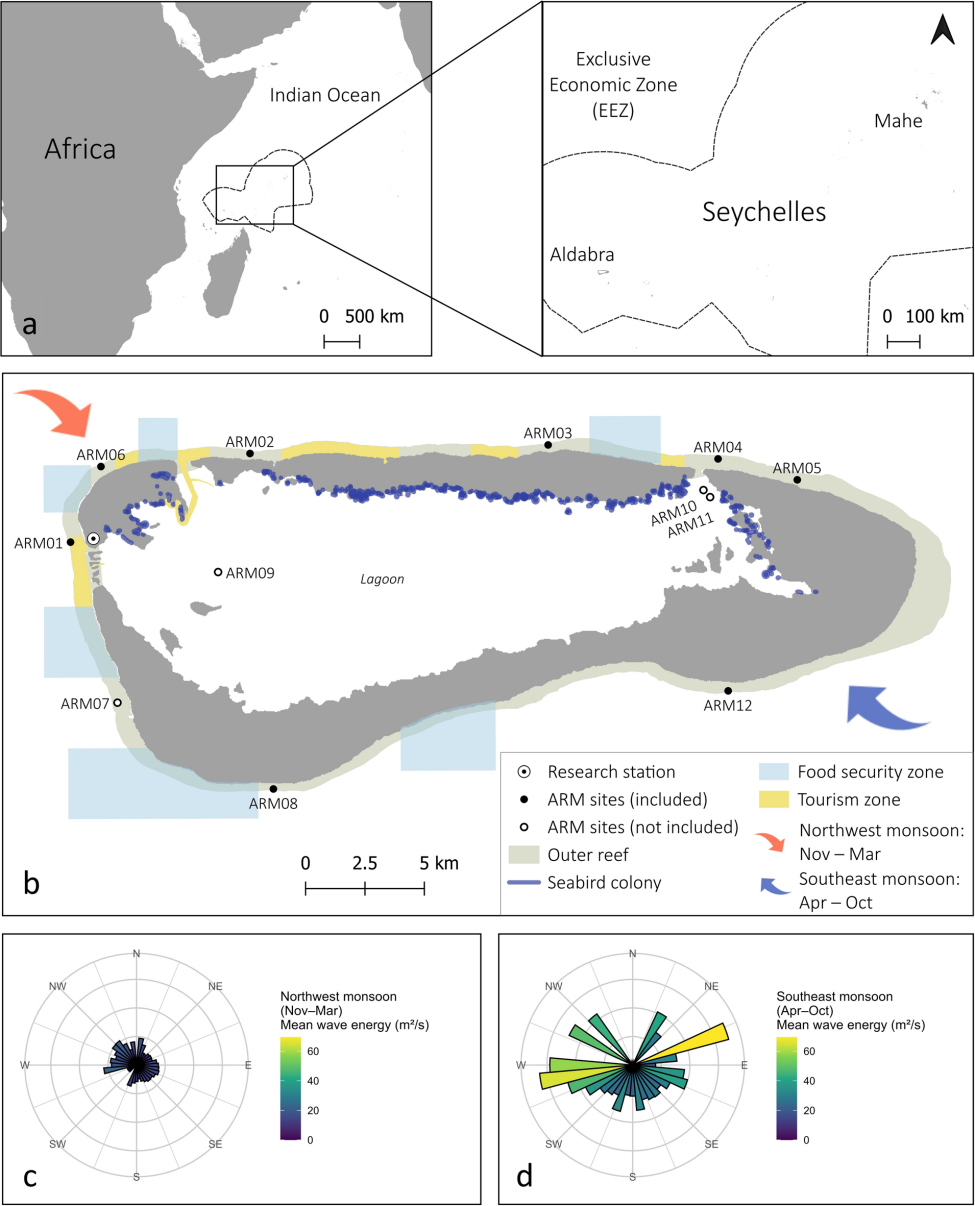
(**a**) Location of Aldabra Atoll in the Indian Ocean and within Seychelles. (**b**) The twelve Aldabra Reef Monitoring (ARM) sites are located on the seaward reef (nine) and inside the lagoon (three) and data from sites 1–6 and 8 is included in the analysis. Blue polygons on the seaward reefs demark zones within which SIF permits handline bottom fishing of its subsistence fishery; yellow zones demark areas within which tourism activities are allowed (snorkeling, diving, boating). Mean wave energy across cardinal directions during the northwest monsoon (**c**) and southeast monsoon (**d**) calculated from satellite data (WaveWatch III Global Wave Model^96^; 50 km resolution) recorded at one raster cell at the southwest of Aldabra: −9.5, 46.0 (also see Methods S1 in the Supplementary Material). Figure modified after Koester et al.^51^ with the map layout adapted from J. Letori’s original design.

Aldabra, a UNESCO Marine World Heritage Site, is a remote atoll in the western Indian Ocean within the Republic of Seychelles, located ca. 400 km northwest of Madagascar (Fig. 1). Aldabra hosts the largest reef system in Seychelles, which, due to its protection and remoteness, has limited anthropogenic stressors. The atoll has been managed by a public trust, the Seychelles Islands Foundation (SIF) since 1979, was designated a Special Reserve in 1981 under the Seychelles National Parks and Nature Conservancy Act, and inscribed by UNESCO in 1982. Aldabra is uninhabited except for 10–20 SIF staff based at the research station, who monitor the atoll’s ecosystems and biodiversity year-round. Ongoing management efforts on Aldabra include maintenance of the no-take protected area (2559 km^2^ since^2^ 2018; formerly 439 km^2^) while allowing limited tourism and a small-scale subsistence fishery, and restoring ecosystems (e.g. invasive alien species eradications^35^; native species re-introductions^36^; marine debris removal^37^). Due to Aldabra’s remoteness and history of protection, its coral reefs are thought to be relatively resilient, with potential to advance knowledge of natural ecosystem variation in the absence of significant direct stressors^38^. Aldabra is also thought to be an important coral larval source for the region^39,40^. The prevailing source of wave energy meeting Aldabra’s coral reefs is wind driven swell from the Indian Ocean monsoonal trade winds^41,42^. This shifts between the shorter wet northwest-dominated months (November to March) to the dry southeast monsoon months (April to October)^43^ (Fig. 1). Average wind speeds are generally higher during the southeast monsoon, leading to rough seas and increased mean wave exposure along the southern and eastern coastlines. In contrast, the northwest monsoon typically brings calmer seas, but sporadic strong winds, associated with passing tropical storm or cyclones, resulting in higher maximum wave exposure along the northern and northwestern coastlines^44^.

### Data collection and processing

Coral reef monitoring projects have been conducted sporadically at Aldabra since the 1960s with varied methodologies^45–49^ but a long-term annual reef monitoring programme was established by SIF in 2014. The 12 permanently fixed Aldabra Reef Monitoring (ARM) sites cover each of Aldabra’s coastlines (Fig. 1), and annual monitoring includes data on fish (a subset of species) and benthic communities, collected along permanent transects^1,38,50,51^ (Table S1). In December 2015, alongside the standard reef monitoring, additional surveys were conducted to obtain data on reef structural complexity, coral recruit density, total fish biomass and total herbivore biomass to supplement existing data (Table S1).

We assessed reef resilience and future management strategies for the Aldabra reef monitoring sites using data collected on eight of the ARM sites in 2014 (percent framework corals) and 2015 (structural complexity, total fish biomass, total herbivore biomass, coral recruit density; Table S1). We selected these data sets as resilience indicators specifically because they had associated published thresholds (established at multiple scales) that we could compare to our site-level data. We were also limited by the scope of our management resources, to only including resilience indicators for which we were able to collect robust spatial and temporal data. This necessarily excluded certain indicators which would have potentially been useful in the context of Aldabra, for example, wastewater pollution^26^ and nutrient regime^27^. The ecological significance of the selected indicators is outlined in Table 1.

At each of the eight sites, three 50-m long transects were laid perpendicular to the reef slope at 5 m depth, with one additional 50-m long transect at 15 m depth.

The fish community was surveyed by recording number, identity (to species) and size (to nearest cm) of all fish ≥ 8 cm by a single observer following Graham et al.^27^ (which builds on Jennings et al.^52^) along the entire 50-m transects with a transect width of 4 m. Fish < 8 cm in size were not recorded to reduce surveyor bias, as high abundances of small fish can reduce consistency in survey methodology and data^50,53^. As resource limitations prevented the implementation of targeted surveys tailored to small-bodied fishes, no data of fish < 8 cm were collected. At the start of every survey day, fish size estimation was calibrated using PVC pipes of known lengths. Count data was converted to biomass (kg/ha) using published length-weight relationships^54–60^. Structural complexity was visually estimated using the 6-point scale of Wilson et al.^61^ at the start of each transect (4 replicates per site). The number, identity and size of juvenile coral colonies (< 5 cm diameter) was recorded in five replicate 0.25-m^2^ quadrats per transect.

Benthic cover was assessed using benthic photoquadrat surveys. A GoPro camera attached to a 70 × 50 cm PVC frame at 70 cm height was used to photograph two 10 m sub-transects on both sites of the permanent 50 m transect (10 m gap between sub-transects) as described in Koester et al.^38^. Approximately 35–43 photos were taken for each sub-transect, and each photo covered 0.35 m^2^ of reef. The photos were analysed using Coral Point Count with Excel extensions^62^ by identifying the benthos at 16 randomly assigned points per image as described by Cerutti et al.^1^.

To contextualise the resilience assessments with observed coral cover trajectories post-bleaching, we utilised coral cover data collected in 2016 and 2022 using the benthic photoquadrat method as described above, with the difference that it has been collected along three 10 m sub-transects (not two as in 2014).

### Study approach and data analysis

All statistical analyses were conducted using R (version 4.1.2^63^). Acknowledging that depth is an important factor influencing reef communities^64,65^ data from the three 5 m and one 15 m transects were pooled as only one transect was surveyed at 15 m depth at each site (see ‘Data collection and processing’). Aldabra’s permanent monitoring sites were established in 2013 at 5 m depth, prior to Graham et al.^27^ identifying 6.6 m as the minimum threshold depth, where shallower reefs are less likely to rebound to pre-bleaching coral cover. The primary (replicated) surveys at 5 m depth fall below this threshold and are therefore considered non-resilient by that indicator. Pooling the transects allowed analyses to be weighted by depth, enabling consideration of the presence of deeper reef communities (see Figure S1 showing all data plotted with and without the 15 m depth transect included).

#### Comparisons with published recommended thresholds

Values of four reef characteristics (total reef fish biomass [including only reef fish following MacNeil et al.^28^, herbivorous fish biomass, coral juvenile density and structural complexity) were compared against reference thresholds from MacNeil et al.^28^ and Graham et al.^28^ with two-tailed one sample t-tests (Table 1), investigating whether each site is significantly different from the recommended value (which has no variance). Given the small sample sizes (3 degrees of freedom), we also undertook permutational analyses to improve our confidence in the outcomes of the t-tests (see Methods S2 in the Supplementary Material). Where results differed between the two approaches, the permutational results were prioritised.

T-test outcomes for each reef characteristic were scored for Aldabra overall and for each site. A score of 1 was given if the observed mean was significantly higher than the reference threshold; zero if the observed mean was not statistically different from the reference threshold; and −1 if the observed mean was significantly lower than the reference threshold (i.e., target was not met).

We combined the different reef characteristics into a ‘composite index’ (see also Maynard et al.^20^ and Gudka et al.^13^) by normalising the sum ($\:\sum\:$) of individual scores (*u*) for each of the four characteristics (*tfb*: total fish biomass, *hbm*: herbivorous fish biomass, *juv*: coral juvenile abundance, *rug*: rugosity) to scale between 0 and 1, giving equal weights across indicators (also see Methods S3 in the Supplementary Material):

$$\:I=\left(\frac{\sum\:\left({u}_{tfb},{u}_{hbm},{u}_{juv},{u}_{rug}\right)-min\left(\sum\:u\right)}{max\left(\sum\:u\right)-min\left(\sum\:u\right)}\right)\times\:100$$

where the minimum possible score is −4 (a site did not achieve the target across all four reef characteristics), and the maximum possible score is 4 (a site exceeded the target across all four reef characteristics).

The composite index therefore provides us with a score out of 100 that is reflective of the site’s potential resilience based on achieving nominated thresholds across all four characteristics (and noting that a fifth, depth, is considered implicitly given the pooling of transects).

#### Contextualising reef resilience assessments with observed post-bleaching coral trajectories

To place the resilience assessments in the context of the actual observed changes in live hard coral cover following the 2015 bleaching event, we calculated (1) the percent change in hard coral cover between 2014 (pre-bleaching) and 2016 (post-bleaching) as a measure of bleaching impact and (2) the percentage of 2022 hard coral cover relative to 2014 levels to assess the extent of recovery towards pre-bleaching state. Given the small sample size, we did not apply formal statistical analysis (e.g., regression analysis or modelling approaches) to evaluate the predictive power of the resilience scores. Instead, we present these results descriptively to provide ecological context for interpreting the site-level resilience assessments.

#### Trophic pyramid structure comparisons

Following the method outlined by Graham et al.^27^ and using fish biomass data (kg/ha) we: (1) assigned trophic levels at the species level using Fishbase^58,66^; (2) calculated relative distribution of biomass among five trophic levels to create trophic pyramids for Aldabra overall and each site individually; and (3) compared these trophic pyramids to the ‘fished’ and ‘unfished’ seascapes of Graham et al.^29^ and the classical trophic pyramid (see Trebilco et al.^67^). The five trophic levels, as suggested by Graham et al.^29^ are: 1: 2.0–2.5 (broadly detritivores and herbivores), 2: 2.5–3.0 (broadly omnivores that tended to herbivory), 3: 3.0–3.5 (broadly omnivores that tended to carnivory, including corallivores and mid-level invertivores and planktivores), 4: 3.5–4.0 (broadly high-level carnivores and invertivores) and 5: 4.0–4.5 (mostly piscivores). To enable direct comparison with Graham et al.^29^ we excluded any fish families that are not part of the 17 target families listed by Graham et al.^29^.

#### Management strategy classification

We used pre-bleaching percent coral cover data (from 2014) and degree heating weeks (DHW) data (2014–2017) accessed from the National Oceanic and Atmospheric Administration^68^ to evaluate the ecological condition of Aldabra’s reef sites and their exposure to climate change following Darling et al.^30^ (Table 1). We then classified Aldabra’s reef sites based on the ‘protect’, ‘recover’ and ‘transform’ management strategies outlined by Darling et al.^30^. These strategies were to: (1) ‘protect’ functioning reefs that were associated with limited exposure to recent bleaching-level thermal stress (DHW < 4 °C-weeks) and maintained coral cover above 10%; (2) ‘recover’ reefs that have recently maintained cover above 10% but were exposed to potential bleaching-level thermal stress (DHW > 4 °C-weeks) during the 2014–2017 global coral bleaching event; and (3) for reefs with coral cover below net-positive carbonate budgets (< 10% hard coral cover), societies may ultimately need to ‘transform’ away from reef-dependent livelihoods^30^.

Ecological condition was represented by the potential for a net-positive carbonate budget before the 2014–2017 bleaching event^30^, one of the worst on record to date^4,34^. To assess ecological condition we: (1) assigned life history strategies (‘competitive’, ‘generalist’, ‘stress-tolerant’, ‘weedy’) at the genera and growth form level following Darling et al.^69^ using the Coral Traits Database^70^; (2) calculated percent cover of (summed) competitive and stress-tolerant corals (hereafter, ‘framework’ corals); and (3) compared Aldabra data against the threshold value (10% cover) using two-tailed one-sample t-tests (and additional permutational analysis as explained above and in Methods S2).

Climate change exposure was represented by thermal stress during the 2014–2017 bleaching event^30^. To assess climate change exposure we: (1) extracted daily DHW data available from NOAA Coral Reef Watch^68^ for the rectangular region surrounding Aldabra, defined by two bounding coordinates (northwest: −9.325, 46.175; southeast: −9.525, 46.525; 35 raster cells; Figure S2) for the period January 2014 to December 2017 through the ERDDAP data server via the Pacific Islands Ocean Observing System (PacIOOS)^71^; (2) calculated the maximum DHW value for each 5 × 5 km raster cell nearest to each survey site for each year (Figure S2); and (3) compared these values against the established threshold for thermal stress exposure (4 DHW °C-weeks^30^).

The two indices (ecological condition and climate change exposure) were then placed into Darling et al.’s^30^ management strategy portfolio, which recommends a ‘protect’ strategy if reefs did not experience thermal stress above 4 DHW (°C-weeks) during 2014–2017 and had > 10% cover of framework corals, a ‘recover’ strategy if reefs were exposed to at least moderate bleaching-level stress (DHW > 4 °C-weeks during 2014–2017) and had > 10% cover of framework corals, and a ‘transform’ strategy if reefs had < 10% cover of framework corals.

## Results

### Recommended threshold comparisons and post-bleaching coral trajectories

Our results suggest high resilience on Aldabra’s seaward forereef slopes. At the atoll level considering all eight sites, observed values for four key reef characteristics met or exceeded recommended thresholds (Table 2). Structural complexity was not significantly different from the recommended threshold (*t* = −0.13125, *df* = 31, *p* > 0.05; 99%CI from permutation tests overlapped with the threshold; Table S2, Figure S3), while total and herbivorous fish biomass and juvenile coral colony density were significantly greater than threshold (*t* = 3.3138, *df* = 31, *p* < 0.01; *t* = 3.8482, *df* = 31, *p* < 0.01, *t* = 5.5305, *df* = 30, *p* < 0.001, respectively; 99%CI from permutation tests did not overlap with the threshold; Table S2, Figure S3). Combining the individual indices into a composite resilience index gave an atoll-level score of 87.5%; on average, Aldabra’s reef exceeded three of the four thresholds (Table 2).

**Table 2:** Unique status assessments considering four reef characteristics (total fish biomass; herbivorous fish biomass; juvenile coral density; structural complexity) overall and at each site. Numbers represent outcomes from the permutation analyses based on two-tailed one-sample t-tests (pooling three ‘5 m depth’ and one ’15 m depth’ transects: df = 3 at the site-level; df = 31 overall; interpreting significance based on 99% confidence intervals); observed mean was significantly higher (1), lower (−1) or not statistically different (0) from the recommended threshold. Last column represents assessment outcomes using the ‘composite resilience index’ expressed on a scale of 0–100%.

Site	Total fish biomass	Herbivorous fish biomass	Juvenile coral density	Structural complexity	Composite index score (%)
Overall	1	1	1	0	87.5
ARM01	0	1	1	1	87.5
ARM02	1	1	1	1	100
ARM03	0	1	1	0	75
ARM04	1	1	1	0	87.5
ARM05	0	1	1	−1	62.5
ARM06	1	1	1	0	87.5
ARM08	0	1	1	−1	62.5
ARM12	1	1	1	−1	75

**Fig. 2:**
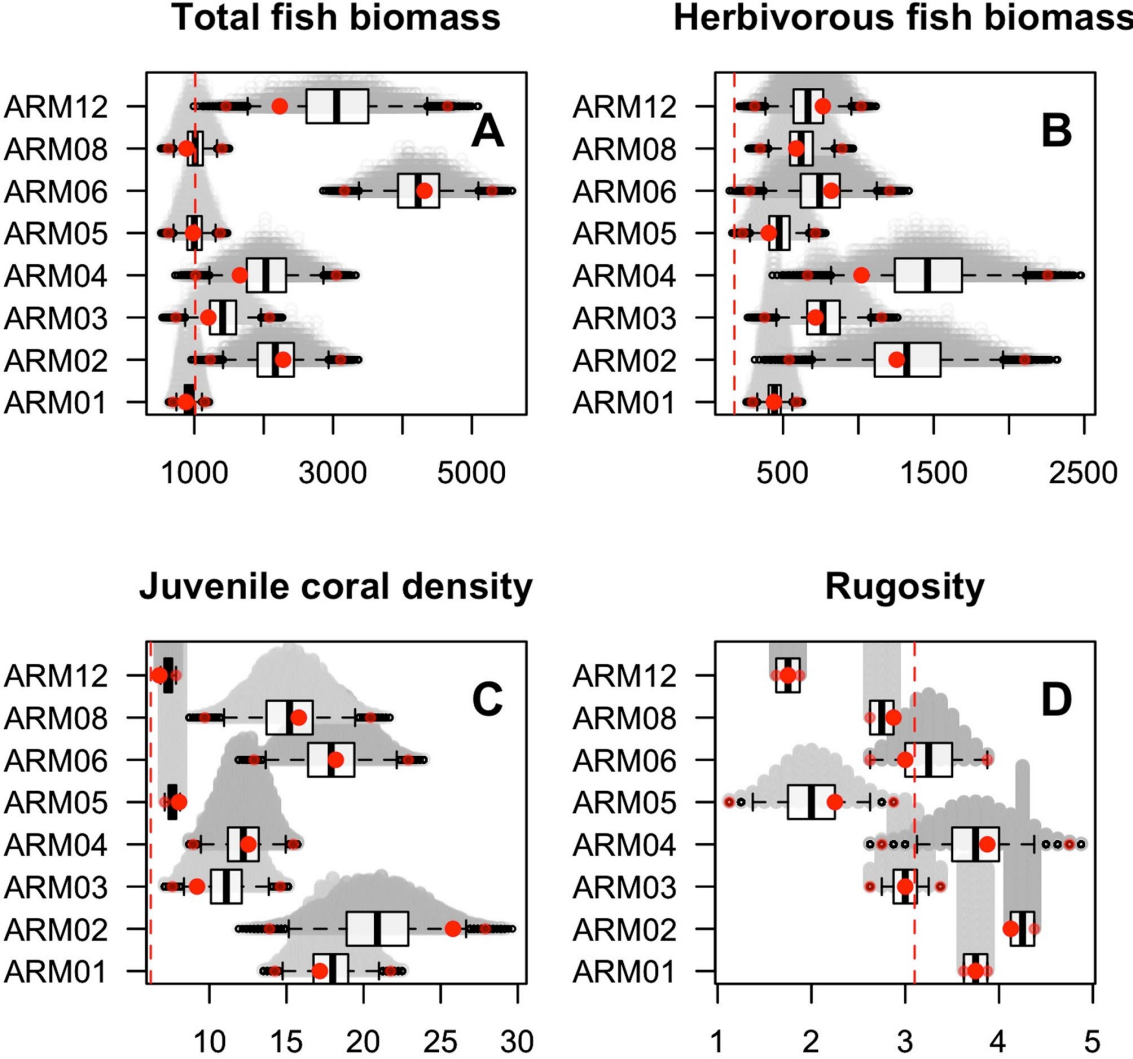
Distributions of the pseudo-sampling from site-level sample populations based on equal probability of pulling values (*n* = 4 samples per pseudo-sample; *N* = 9999 permutations) from the range of transect-level values for each of the four reef characteristics (mean estimate) overlaying stacked strip charts (pale grey circles) to show distribution of the pseudo-samples. Black vertical bars represent the 25th percentile, median, and 75th percentile from left to right. Whiskers extend to the largest value within 1.5 times the interquartile range (IQR) above the 75th percentile and the smallest value within 1.5 times the IQR below the 25th percentile. Dotted red line shows the theoretical threshold (a: MacNeil et al.^28^; b-d: Graham et al.^27^), large red circles show the observed means, small red points show the 0.005 and 0.995 quantiles. We interpret a significant difference between the indicator and the recommended threshold when one or both quantiles do not overlap with the threshold value.

When investigating individual sites, three (of the eight) sites did not meet the threshold for structural complexity but thresholds were met or exceeded for the other three reef characteristics across all sites (Table 2; Fig. 2, Table S2). Composite resilience index scores were above 60% across all eight sites (Table 2, Table S2). Four sites with an index score of > 80% (ARM01, ARM02, ARM04, ARM06) showed variable bleaching impacts (34.0–72.6% hard coral cover loss post-bleaching) but had recovered to 62.4–88.7% of their pre-bleaching cover by 2022 (Table 3; Figure S4, and S5). Two sites with the lowest composite resilience index scores (both 62.5; ARM05 and ARM08) had disparate recovery trends, regaining 42.8% and 94.4%, respectively, of their pre-bleaching cover by 2022 despite bleaching-related declines of 61.3% and 48.3% (Tables 2 and 3; Figure S4 and S5). Framework corals at ARM05 made up 24.4% of total coral cover compared to 67.5% of total coral cover at ARM08 (Figure S6, Table S4). Similar to ARM05 and ARM08, site ARM12 did not meet the structural complexity threshold and had the lowest pre-bleaching coral cover (9.7%; Tables 2 and 3, Figure S5). However, framework corals made up 75% of that cover, mostly stress-tolerant species, and recovery post-bleaching was 100% in 2022 following minimal bleaching impact (14.4% reduction in coral cover; Tables 2 and 3, Figure S6, Table S4).

**Table 3:** Resilience assessment (composite index) and management assessment in relation to the absolute percentage hard coral cover before (2014) and after (2016, 2022) the 2015/16 coral bleaching event at Aldabra and the relative changes following the event.

Site	Composite index score	Management assessment	Pre-bleaching (2014)		Post-bleaching (2016)		Post-bleaching (2022)		Bleaching impact ^a^	Recovery^b^
			Mean	SE	Mean	SE	Mean	SE		
Overall	87.5	Recover	22.5	2.3	10.4	0.9	17.0	1.6	−53.8	75.6
ARM01	87.5	Recover	26.8	1.9	12.2	2.1	16.7	2.7	−54.4	62.4
ARM02	100	Recover	34.1	7.5	9.3	1.9	22.3	5.4	−72.6	65.5
ARM03	75	Recover	14.2	5.4	7.8	1.6	15.2	4.2	−44.7	107.0
ARM04	87.5	Recover	29.4	6.0	10.0	1.4	17.9	3.7	−65.9	60.9
ARM05	62.5	Transform	12.3	6.9	4.8	1.8	5.3	2.0	−61.3	42.8
ARM06	87.5	Recover	28.1	7.6	17.6	4.6	24.9	5.7	−37.5	88.7
ARM08	62.5	Recover	25.5	4.0	13.2	1.0	24.1	3.5	−48.3	94.4
ARM12	75	Recover	9.7	1.4	8.3	0.9	9.7	1.1	−14.4	100.0

^a^Relative change in percentage hard coral cover between 2014 and 2016.

^b^Percentage hard coral cover in 2022 as percentage of hard coral cover in 2014.

Site ARM02 exceeded thresholds for all four of the reef characteristics (Fig. 2), giving a composite resilience index score of 100% (Table 2) yet also lost the highest proportion of coral cover during the bleaching event (−72.6%; Table 3). Framework corals made up 66.8% of the site’s 34.1% coral cover in 2014 (Figure S6, Table S4), and competitive species specifically accounted for 50.6% of the total cover. By 2022, total coral cover had regained 65.5% of its pre-bleaching levels (Table 3).

### Trophic pyramid structure comparisons

**Fig. 3:**
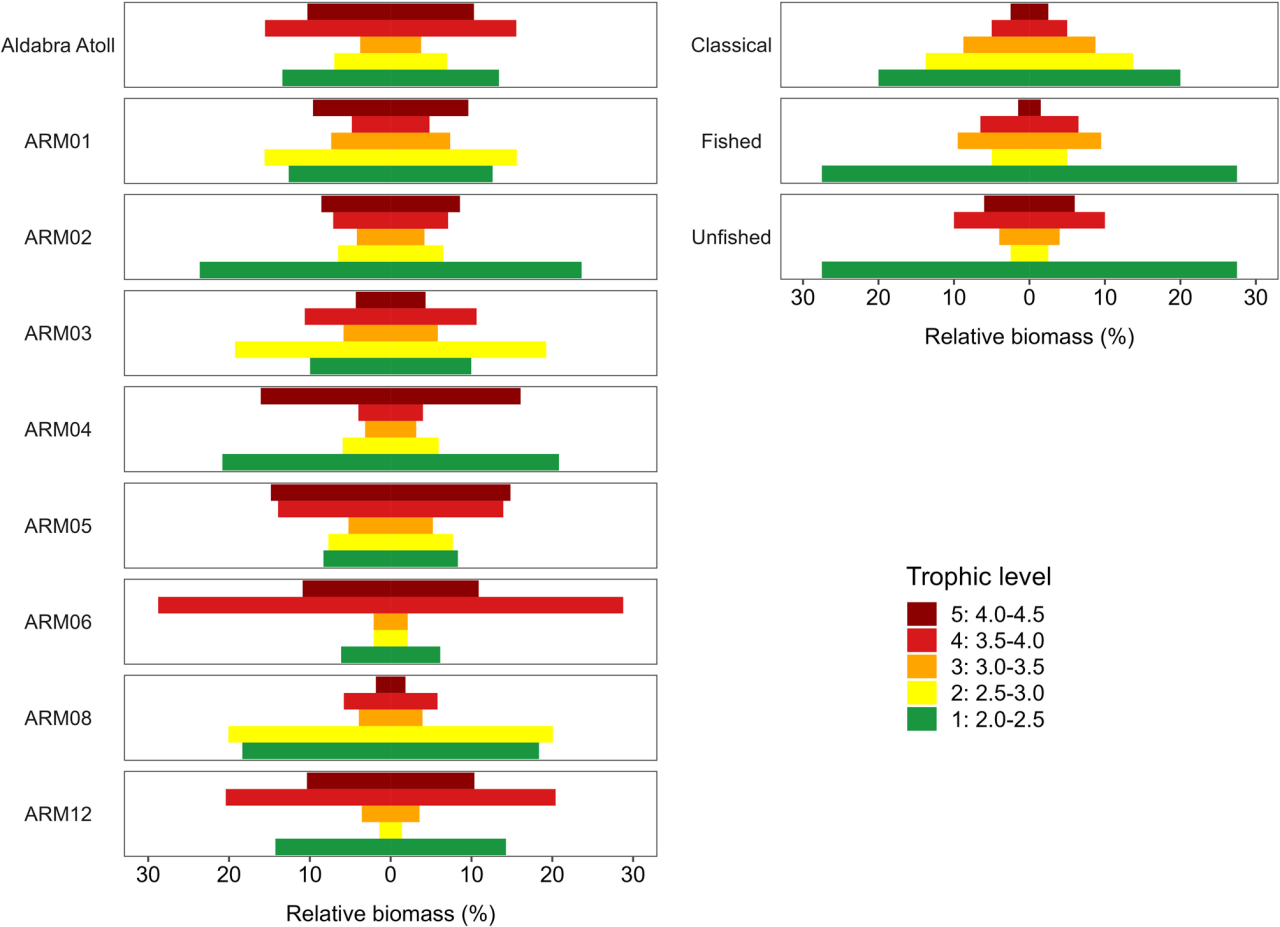
Relative biomass distribution among trophic positions based on species from 14 families of non-cryptic, diurnal, reef-associated fishes. Plots on the left represent results of Aldabra overall and individual sites, plots on the right show comparative pyramids based on the “Classical” expectation^67^ and the expected biomass of fished and unfished locations of Graham et al.^29^ for areas where total biomass is between 665–4915 kg/ha (log biomass 6.5 to 8.5; reproduction of their Figure S2). Colours represent trophic levels: 1: 2.0–2.5 (dark green), 2: 2.5–3.0 (yellow), 3: 3.0–3.5.

The overall trophic pyramid of the reef fish community compared favourably to Graham et al.’s^29^ concave unfished pyramid shape (Fig. 3, Figure S7). At the site-scale, seven of the eight pyramids showed higher proportions of upper trophic level biomass than expected based on a classic biomass pyramid^67^ approaching Graham et al.’s^29^ concave shape. Community biomass was greater than the recommended 650 kg/ha at the atoll-level (mean biomass = 1846 ± 255 SE kg/ha) and for all sites (mean biomass ranged from 986 ± 157 kg/ha at ARM01 to 4267 ± 756 kg/ha at ARM06; Table S2).

### Management strategy classification

All but one of Aldabra’s seaward coral reefs fell within the ‘recover’ strategy of Darling et al.’s^30^ management portfolio: predicted maximum heat stress exceeded 5 DHW in April 2016 at all sites (Table S3, Figure S8) and the cover of competitive and stress-tolerant coral species did not differ from the 10% threshold at all sites except ARM05 (Table S4; Figure S9). Site ARM05 therefore fell within the ‘transform’ strategy, whilst all other sites fell within the ‘recover’ strategy.

## Discussion

In the absence of local resilience thresholds, we assessed the site-level resilience of Aldabra Atoll’s seaward reefs using multiple broadscale threshold approaches. We found that in 2015—prior to the mass bleaching event—Aldabra’s seaward reefs exhibited resilience characteristics consistent with those of a well-managed, remote marine reserve. All eight sites surveyed met or exceeded thresholds for several key resilience indicators, including total and herbivorous fish biomass, trophic fish biomass distribution and juvenile coral density. Three sites did not meet the resilience threshold for structural complexity but composite resilience index scores were above 60% across all eight sites. Under Darling et al.’s^30^ management strategy framework, all but one site were classified within the “recover” category, indicating strong potential for recovery despite experiencing severe bleaching. Notably, the resilience predictions closely matched observed post-bleaching coral cover at Aldabra at most sites (Table 3), which highlights the value but also the limitation to the application of these approaches.

### Resilience assessment

**Fig. 4:**
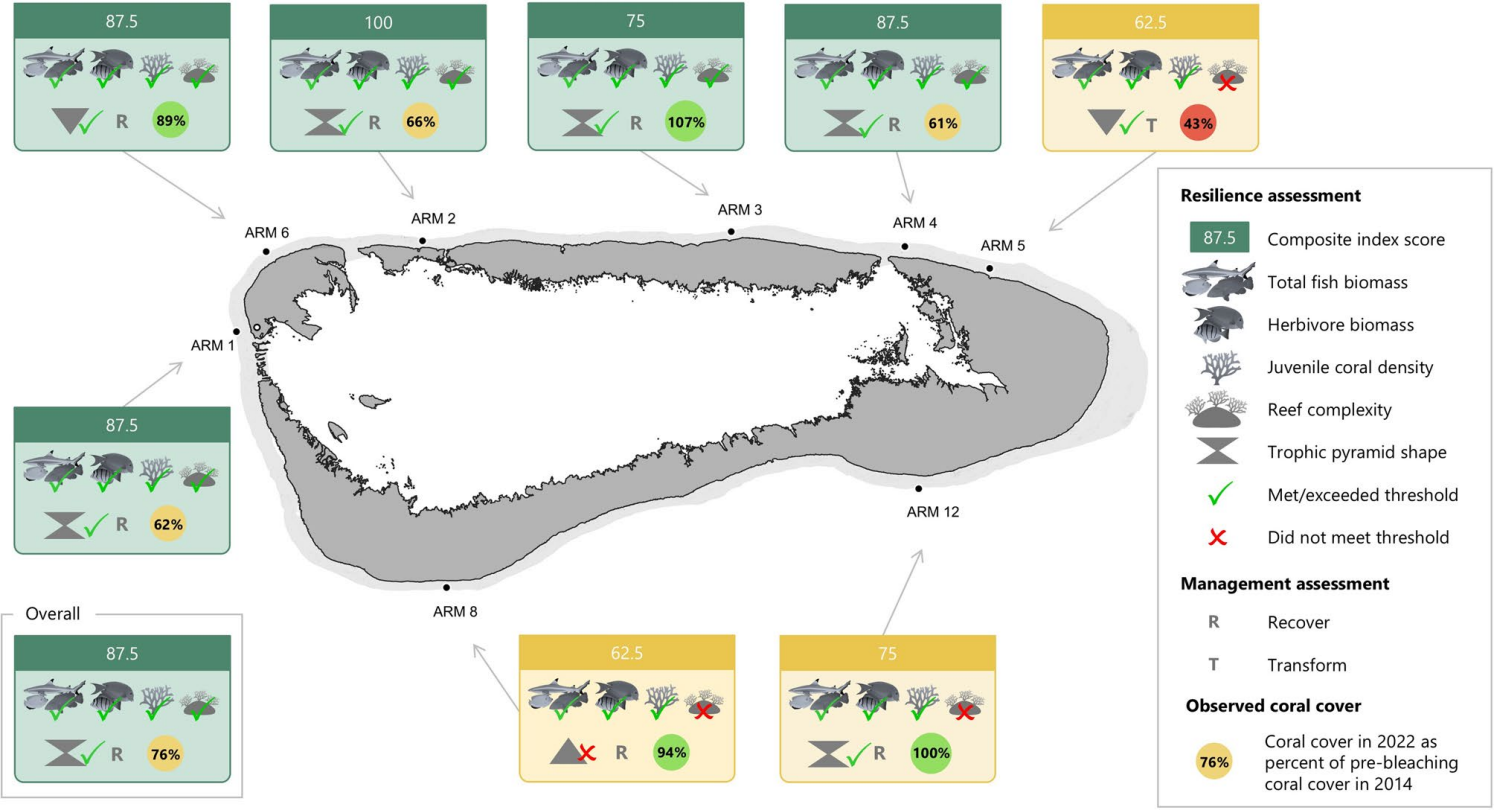
Visualisation of results from the resilience assessment and future management assessment overall (inset) and at each site. Green assessment cards indicate all thresholds were met or exceeded; yellow assessment cards show that at least one threshold was not met.

In 2015, fish biomass at all seaward coral reef sites on Aldabra met or exceeded recommended thresholds (total fish biomass: 1000 kg/ha^28^; herbivore biomass: 177 kg/ha^27^; Fig. 4), with total biomass ranging from 986 to 4267 kg/ha and herbivore biomass from 264 to 958 kg/ha. While comparisons between reef locations should be approached cautiously due to differences in biophysical drivers like primary productivity^25,28^ Aldabra’s biomass levels are broadly comparable to other remote, protected areas such as the Chagos Archipelago^72^. Notably, Aldabra’s average total biomass more closely resembles that of Diego Garcia—an inhabited atoll with subsistence fishing—than the unfished, uninhabited Northern Atolls. This could indicate that even Aldabra’s low human density (< 1 person/km²) may influence fish biomass through subsistence fishing, although not to a level that is deemed problematic.

Fishing pressure mapping from 2005 to 2015 showed the highest pressure was near the Research Station on Aldabra’s northwest coast and the lowest along the east coast^44^. Our finding show that even within the most heavily fished area, biomass ranged from 986 to 4267 kg/ha, the latter being the highest recorded across surveyed sites. Our results indicate that current levels of subsistence fishing are having limited impacts on current overall biomass, though the lack of historical fish biomass data limits definitive conclusions.

Supporting this, Aldabra’s fish biomass exhibits a concave trophic pyramid structure (except at ARM08), with most biomass concentrated in the highest (predators) and lowest (herbivores) trophic levels. These pyramids reflect energy-balanced communities^29^ and are similar to other remote and protected locations (e.g. Mexico^57^ and in the central Pacific^25^. However, a limitation in the dataset we used and a factor influencing the trophic pyramids, is the exclusion of small fish, which were omitted to minimise surveyor bias (as was also the case in Graham et al.^29^). This exclusion likely led to an underestimation of biomass in lower trophic groups (e.g., planktivores, omnivores, microinvertivores) and cryptobenthic taxa, which can substantially contribute to overall reef fish productivity^73,74^.

Despite the current resilience of Aldabra’s fish communities, future health is threatened by climate change impacts on fish biomass and community structure^75–77^ as well as increasing poaching activity, particularly farther from the Research Station (SIF, unpublished data). As fishers venture farther and stocks decline elsewhere, Aldabra becomes increasingly attractive, greater surveillance and enforcement of the reserve will be a key management strategy to continue to meet these resilience thresholds.

In 2015, SIF established and began enforcing six designated fishing zones to monitor and manage fishing pressure and to facilitate long-term tracking of biomass changes in the absence of fishing. To mitigate potential impacts, the Aldabra subsistence fishery could shift focus from bottom fishing (targeting reef fish) to pelagic trolling, or set total catch limits.

The high herbivorous fish biomass found across Aldabra’s reef sites is a factor which promotes coral recruitment by maintaining available substrate for coral larvae settlement^27^. We found that coral juvenile density at all reef sites in this study (7.0–25.8 coral juveniles/m^2^) met or exceeded the recommended resilience indicator threshold^27^ of 6.2 coral juveniles/m^2^. Three of the eight sites did not meet the recommended threshold for structural complexity. The sites are exposed to high and persistent wave energy for extended periods; the southeast monsoon season (April–October)^44^ affects ARM05, ARM12 and ARM08, which are located in the north-east, south-east and south-west of the atoll (Fig. 1b, d), with northwest monsoon (November–March)^44^ also affecting ARM08 (Fig. 1b, c). Reefs under such high wave energy conditions naturally consist of more stable coral growth forms (e.g. encrusting and massive^78^), impacting the structural complexity score of the reef. The three sites met all other thresholds, highlighting site-specific factors that can now be considered and incorporated when refining the local thresholds used for Aldabra’s RBM.

### Predicted resilience vs. post-disturbance trajectories

Our 2015 resilience assessment presents a snapshot of one point in time, but studies of Aldabra’s reefs from as early as the 1960 s describe the north-west coast as having the most ‘luxuriant coral growth’^48,49^. These descriptions correspond with our 2015 findings of high structural complexity, a net positive carbonate budget, and the high resilience index scores at sites on the north-west coast. This, albeit anecdotal evidence, demonstrates the long-term resilience of several areas of reef, despite the impact of the 1998 global coral bleaching event^79^. A more rigorous comparison was made using contemporary coral cover data, collected annually at Aldabra since 2014, and which spanned the 2016 bleaching event. The event peaked at Aldabra in April 2016, causing an overall reduction in relative hard coral cover of 54%^1,38^, with substantial variation across sites (14–73% reduction). By early 2022, six years post-bleaching, hard coral cover at these sites reached 43–107% of the pre-bleaching cover. This highlights that live coral recovery (one component of ecosystem resilience) varied across sites, and in some cases did not align with our estimates of predicted resilience. For example, our 2015 resilience assessment highlighted ARM08 as having one of the lowest composite resilience scores, primarily driven by low structural complexity. This site lost 48% of its coral cover in 2016, but regained 94% of its pre-bleaching coral cover, indicating fast recovery to greater than 20% live coral cover, a recognised threshold^80,81^. Considering the proportion of framework corals provides added insight^30^: almost 50% of its pre-bleaching coral cover was made up of stress-tolerant species, likely driving the potential resistance to the thermal stress, and 20% of its pre-bleaching cover was made up of competitive species, which may have kick-started recovery. This further highlights the need for a more locally-relevant structural complexity threshold.

Conversely, site ARM05, which also had one of the lowest composite resilience scores driven by low structural complexity, experienced relatively limited hard coral recovery, only reaching 43% of the pre-bleaching cover by 2022. Framework corals made up only 24% of its pre-bleaching coral cover of 12%, which was one of the lowest around the atoll in 2014, putting it into the ‘transform’ management strategy. The lower recovery trajectory may also be attributed to rapid expansion of the calcareous green algae *Halimeda* spp. after the 2016 coral mortality event, which is more abundant on the eastern seaward reefs of Aldabra^38^. Since the 1970s, the greater dominance of *Halimeda* in the east has been attributed to an increase in hydrodynamic energy from west to east^45,82^. While oceanographic differences across Aldabra’s coastlines still need in-depth investigation, recent isotopic analysis of *Halimeda* samples from these reefs shows ARM05 to have the highest level of seabird derived nutrients (SIF unpublished data). *Halimeda* is known to benefit from nitrate and phosphate enrichment, both from upwelling^83,84^ and seabird guano sources^85^ which could be a factor in the *Halimeda* dominance on this reef. This highlights the many reef-level factors influencing reef recovery that are not captured by our resilience assessment^86^ and that the development of specific thresholds of *Halimeda* and crustose coralline algae cover as metrics of recovery would likely be beneficial for future resilience assessments.

Another site-specific factor to consider is Aldabra’s daily temperature regimes whereby Aldabra’s lagoon coral reefs experience much greater daily temperature fluctuations than the outer reefs^38^. Such high-frequency temperature variability (i.e., daily temperature range) is an influential factor in predicting bleaching prevalence and can have a mitigating effect, such that increases in daily temperature range reduce the odds of more severe bleaching^87^. Indeed, the lagoon corals suffered less mortality and saw faster recovery from the 2016 bleaching event^38^. These fine-scale thermal dynamics have been detected via in-situ temperature loggers, rather than the DHW data using 5 km scale satellites derived data used in the Darling et al.^30^ management assessment approach, highlighting another limitation to using such methods, albeit we did not include lagoon reefs in our resilience assessment. Aside from nutrient supplied via upwelling zones^88^ localised nutrient input from seabird colonies via guano deposits may also influence the resilience potential of adjacent reefs around islands^89^. At Aldabra these inputs are patchy^90^ due to tidal changes, strong currents and because seabird colonies (Fig. 1) are restricted by the presence of invasive mammals. Both of these factors (fine-scale temperature regimes and levels of seabird-derived nutrients) should be considered as additional broadscale resilience indicators to future RBM assessments, although these proposed indicators require more resources to acquire site-level data than the indicators used in this study.

### Management assessment

All but one of Aldabra’s seaward reef sites in this study fell within the ‘recover’ strategy under Darling et al.’s^30^ approach. The one site that fell within the ‘transform’ strategy was ARM05, which did not meet the threshold of 10% of framework corals in the 2015 pre-bleaching resilience assessment and also had the slowest recovery trajectory post-bleaching. Earlier in the discussion, we acknowledged that oceanographic processes undoubtedly shape reef communities and contribute to the differences between our survey sites. Since we are unable to determine the extent of their influence with the data currently available and because these oceanographic factors are also beyond our control as managers, we focus our discussion below on known human activities and impacts that may also be influencing site-level differences in resilience.

The management goal in the ‘recover’ strategy is to move reefs back above the 10% threshold of framework coral cover as quickly as possible following climate impacts. Management strategies to achieve this could include minimising local stressors (e.g., invasive species control on islands, pollution control, reduced tourism activities, reduced fishing pressure) and conducting active restoration (e.g., coral gardening or other reef restoration techniques, coastal habitat restoration). Two Aldabra reef monitoring sites with high composite resilience index scores (ARM01, ARM06) are located closest to local human activity and potential stressors: the research station, the food security and the tourism zones (Fig. 1). These findings suggest that the current level of human activity in the vicinity of these sites has minimal impact on site-level reef resilience.

Other local stressors identified by managers, that may negatively impact coral reefs, include plastic pollution and invasive alien mammals. An estimated 500 tonnes of plastic pollution have accumulated along Aldabra’s coastline^37^. Evidence suggests that plastic could impact coral health and subsequent resilience^91^. SIF already undertakes active management of this issue through regular clean-ups, although limited resources hinder removal at scale^37^. Invasive alien mammals are known to suppress seabirds and their nutrient inputs on islands^92^. Eradicating introduced rats and cats from Aldabra would kick-start restoration of a more diverse breeding seabird community across the atoll. This in turn is expected to substantially increase the quantity and distribution of seabird-derived nutrient subsidies entering near-shore reef systems. These nutrient subsidies are now understood to enhance reef resilience through faster coral growth, higher recruitment rates and higher reef fish biomass^85,89,92–94^. As a strategy to enhance coral resilience at Aldabra, a rat and cat eradication is therefore of highest priority and an eradication feasibility assessment for the atoll is currently underway.

### Usefulness of the approaches used

As reef managers of the largest reef system in Seychelles, we found the exercise of applying broadscale threshold approaches to instigate RBM at Aldabra, time-consuming but worthwhile. In particular we found the comparison of Aldabra reef-scale data to broadscale resilience indicator thresholds for structural complexity, total fish biomass, total herbivore biomass, coral recruit density, and comparison of trophic structure, more relevant for informing fine-scale management than the allocation of a management strategy approach. This is, in part, because the scale of application is not entirely relevant to Aldabra management actions; for example, even on the ‘transform’ reef there is no action under this strategy that applies to Aldabra (“transform: where societies may need to transition away from reef-dependent livelihoods”^30^). Whereas the individual resilience indicators provide a more nuanced approach; if for example, we had found that resilience scores were lowest near to the research station, we would potentially invest resources in identifying which activities are impacting these and adjust management accordingly. However, the exercise also highlighted limitations of applying broadscale thresholds to Aldabra’s reefs in the absence of data on a vast array of factors influencing resilience and resilience indicators. In an ideal scenario we would collect reef site level data on the oceanographic, biological, and chemical conditions alongside our chosen resilience indicators. However, while marine science at Aldabra is growing, resources continue to be stretched, with limited project funding available for projects that are research focused without tangible management actions as outputs.

Lastly, combining resilience indicators into the composite index was an effective way of summarising multiple results at monitoring site level, but the variability of index scores between sites meant an atoll-level score was less valuable from a site management perspective and more applicable if the approach was scaled-up to categorise the national reef system.

A caveat of the resilience and future management assessment used here is that it does not account for projected future heat stress. It remains uncertain whether reefs that have demonstrated resilience in the past will continue to do so under the increasing frequency and intensity of bleaching events. In Seychelles, severe bleaching is predicted to become an annual event by 2040-2050^95^. Therefore, applying similar resilience-based assessments across Seychelles and other island groups, particularly where data is available from multiple past disturbance events, could help understand the robustness of these assessments and determine if resilience indicators remain valid over time. Such reef resilience mapping could be combined with recent coral reef connectivity data^39,40^ to strengthen and inform reef system management strategies, define national-level resource and conservation priorities and feed into monitoring national contributions to meeting the Kunming-Montreal Global Biodiversity Framework objectives. With the development of appropriate tools, such as analysis and tracking tools via an open access app, RBM approaches are likely to be extremely important for identifying and protecting the reefs most likely to survive repeated bleaching events. With bleaching events becoming more frequent and intense, applying these broadscale thresholds can provide reef managers with tangible resilience targets to maintain, improve or aim for, and help guide and implement adaptive RBM actions for coral reefs.

## Electronic supplementary material

Below is the link to the electronic supplementary material.

Supplementary Material 1

## Data Availability

The data on fish diversity, abundance, and biomass utilised in this research is accessible on Figshare (DOI: https://doi.org/10. 6084/m9.figshare.22792580.v1). The data on coral juvenile abundance and diversity is available in the supplementary files of Koester et al. 2021 (PLOS ONE 16(12): e0260516. https://doi.org/10.1371/journal.pone.0260516). All other data used for this research is available from the corresponding author on reasonable request.
