# Supplementary material for: Supporting resilience-based coral reef management using broadscale threshold approaches
Source: Sci Rep. 2025 Jul 16;15:25836. doi: 10.1038/s41598-025-09531-9 (PMC12267520; doi:10.1038/s41598-025-09531-9)
Supplement: Supplementary file 1 — Supplementary Material 1 [file 41598_2025_9531_MOESM1_ESM.pdf]

Supplementary Material

Supporting resilience-based coral reef management using broadscale threshold approaches

April J. Burt[1a], Anna Koester[1,2a*], Nancy Bunbury[1,3], Philip Haupt[1,4], Rowana Walton[1], Frauke Fleischer-Dogley[1] & Karen M. Chong-Seng[1,5]

[1] Seychelles Islands Foundation, PO Box 853 Victoria Seychelles

[2] Marine Ecology Department, Faculty of Biology & Chemistry, University of Bremen, Bremen, Germany

[3] Centre for Ecology and Conservation, University of Exeter, Cornwall Campus, Penryn, TR10 9FE, UK

[4] Kent and Essex Inshore Fisheries Conservation Authority, Ramsgate, Kent, CT11 9HD, UK

[5] ARC Centre of Excellence for Coral Reef Studies, James Cook University, Townsville QLD 4811 Australia

[a] Joint first author

All tables, figures, and supplementary methods are presented in the order in which they are first referenced in the manuscript.

Table S1: Alterations and additions to standard survey methods of SIF's Aldabra Reef Monitoring (ARM) programme to accomplish the reef resilience assessment presented in this study

Survey	Original ARM method	Change in 2015	Reason for change	Resilience metric concerned
Fish surveys: size estimates	Six size class bins: 0-4 cm, 5-9 cm, 10-19 cm, 20-29 cm, 30-39 cm, 40 cm+	Sizes estimated to nearest cm for all fish counted.	More precise size estimates enable more precise biomass estimates.	Herbivorous fish biomass<br>Total reef fish biomass<br>Trophic level biomass
Fish surveys: species considered	Only indicator species surveyed (84 taxa)	Including all individuals encountered of non-cryptic, diurnally active, reef-associated species	Two metrics used in our assessment require data from the entire diurnal reef fish community. Only including certain species would preclude comparison with known reference values from Graham et al.[1,2] and MacNeil et al.[3]	Herbivorous fish biomass<br>Total reef fish biomass<br>Trophic level biomass
Benthic surveys: rugosity [a]	Not included	Visual estimates along a 5-point scale	Key factor affecting recovery patterns via increased habitat variability for a diverse array of other organisms to inhabit, including niche space for coral settlement and survival[2]	Structural complexity
Benthic surveys: juvenile coral census [b]	Not included	Quadrats within belt transect	Key factor affecting recovery patterns via successful settlement, survival and growth of new corals[2]	Juvenile coral density
Benthic surveys: percent benthic coverage	Benthic photoquadrats along two (2014) or three (since 2016) 10 m long transects per depth	No change, but resilience assessment in this study uses benthic cover data from 2014	Surveys could not be completed in 2015	Percentage of framework corals

[a] part of the annual ARM surveys since 2021

[b] part of the annual surveys since 2015

Methods S1: Calculation of wave energy

Wave energy was estimated using a widely applied relative metric, calculated as the product of the square of significant wave height (H_s) in meters and peak wave period (T_p) in seconds[4]: $E = H_s^2 \times T_p$

The map below shows the raster cells (yellow) for which data on significant wave height (meters), peak wave period (seconds) and peak wave direction was obtained from the WaveWatch III Global Wave Model[5]. The plots from the raster cell in the southwest of Aldabra are shown in Figure 1.

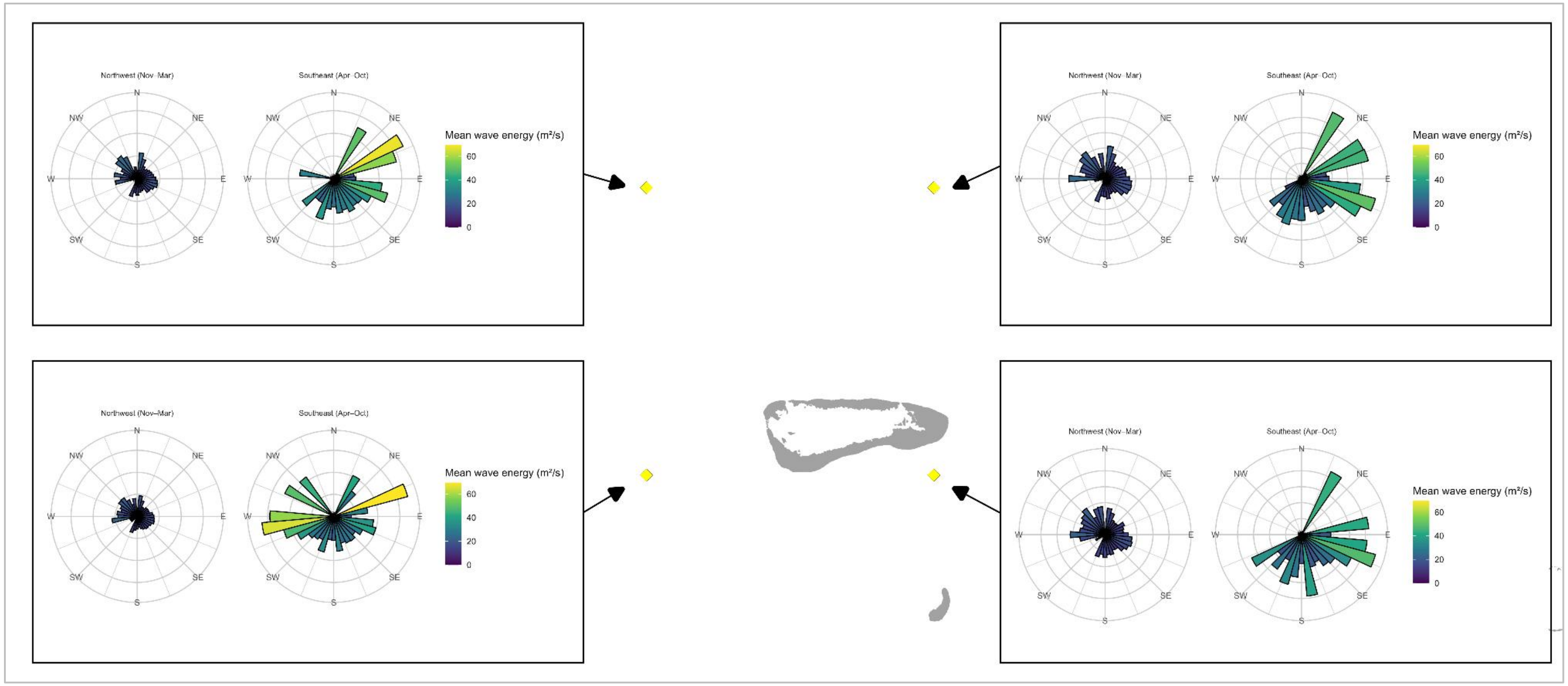

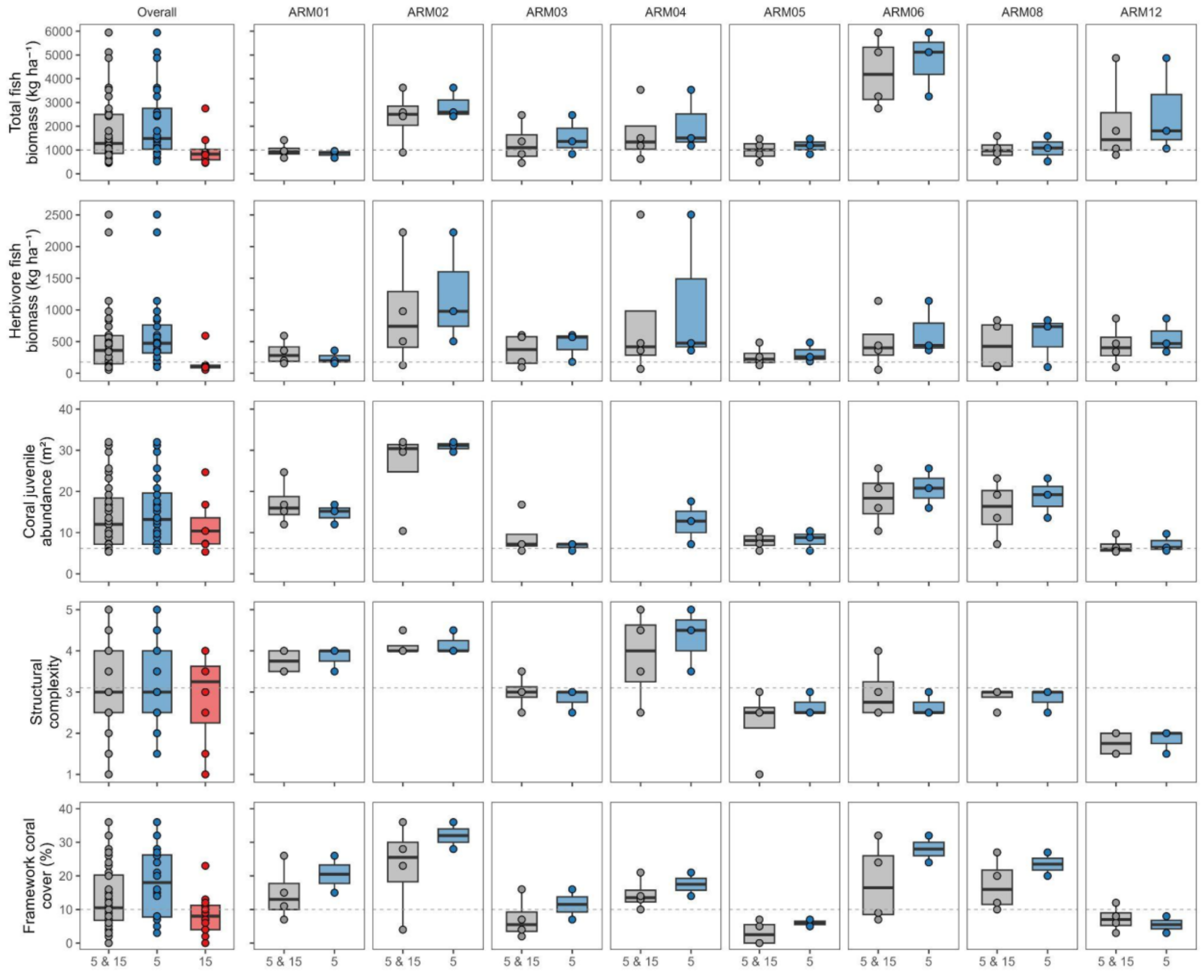

Figure S1: Assessed reef resilience data sets plotted per depth. Plots show median and interquartile range with outliers displayed as dots.

Methods S2: Permutations at each *site* for each reef characteristic

Calculations were done as follows:

1. Four new values were picked, sampling from the range of observed values using base::sample() function.
    a. Sampling was done with no replacement except for the structural complexity reef characteristic ("rug"), which had fewer possible values (6 point scale, but with 0.5 scores accepted: i.e. sequence from 0 to 5 in 0.5 steps).
2. A two-tailed one-sample t-test was calculated.
3. The value of the random mean was extracted and stored (t-test estimate statistic).
4. Steps 1-3 were repeated 9999 times for each variable at each site.
5. 99% confidence intervals were calculated using stats::quantile() function.
6. Results were visualised, focussing on the distribution of random samples in relation to the reference mean (mu).

Permutations at the atoll-level were calculated similarly, except sampling occurred from the full range of observed values for each reef characteristic.

<u>R code to undertake the permutations for each variable at each site</u>

Where:

- *dat is a transect-level dataset of all reef characteristics.*
- *s is each of the eight sites (ARM1-6, 8, 12) .*
- *v is each of the reef characteristics: total fish biomass, herbivorous fish biomass, juvenile coral density and rugosity ("rug").*
    - *v was also run as "cover of stress-tolerant + competitive coral species" and compared against mu=10.*

```
dat <- fulldat[grepl(s,rownames(fulldat)),v]          #or if atoll-level: dat <- fulldat[ ,v]
set.seed(101)
nsim <- 9999
res <- numeric(nsim)
for (i in 1:nsim){
      if(v=="rug") {
          perm <- sample(seq(min(dat),max(dat),0.5),size=4,replace=TRUE)
          while(var(perm)==0) {
                 perm <- sample(seq(min(dat),max(dat),0.5),size=4,replace=TRUE)
      }
                 #Chose replace = TRUE to account for the potentially limited selection of
                 #rugosity values, where duplicates occurred.
   }
      else perm <- sample(min(dat,na.rm=TRUE):max(dat,na.rm=TRUE),size=4)
                 #^i.e. there are only 4 observed values per site (from each transect), so
```

```
                  # sampling any four from the range from that site. Then the t-test:
  tt <- t.test(perm,mu=ref$ref[ref$var==v])
  res[i] <- tt$estimate
                  #^ the value of the random mean is stored
# 99% confidence intervals:
  ci <- data.frame(lw=sapply(res,function(x) quantile(x,0.005)),
                   uw=sapply(res,function(x) quantile(x,0.995)))
```

Methods S3: Differential weighting of indices by the composite resilience index

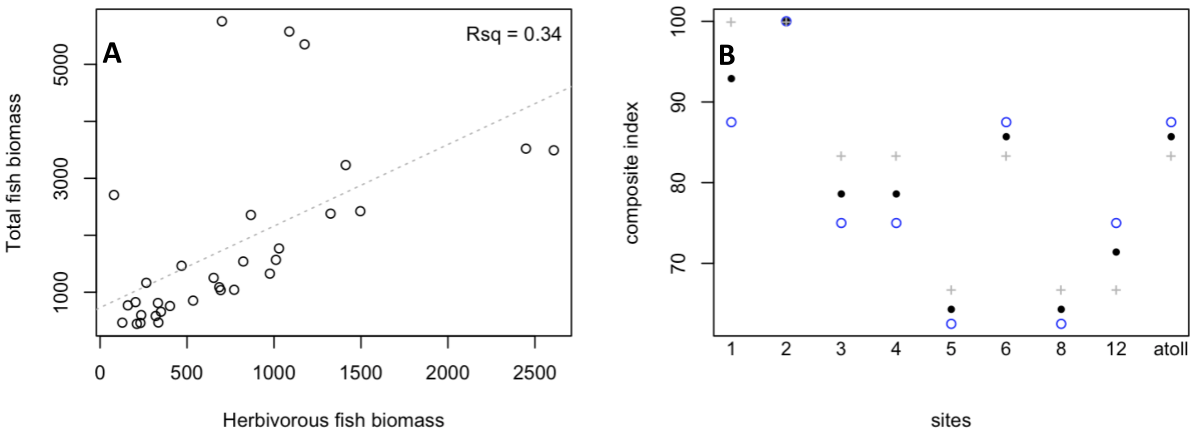

Plot A shows the correlation between two indicators: total fish biomass and herbivorous fish biomass, since necessarily, the latter is captured as part of the former. Total fish biomass was chosen to be down-weighted because herbivory is considered one of the fundamental ecosystem processes that influence resilience.

Plot B shows the influence of weighting on the composite resilience index score at each site (ARM 1-6,8,12) and at atoll-scale. Open blue circles are when all four indicators are assigned equal weights. Filled black points are when the indicator for total fish biomass was downweighed by 0.5 compared to the other three indicators. Grey crosses are when only three indicators are considered and assigned equal weights (excluding total fish biomass).

Down-weighting total fish biomass highlighted more nuanced differences between the sites, but did not really fundamentally change our results.

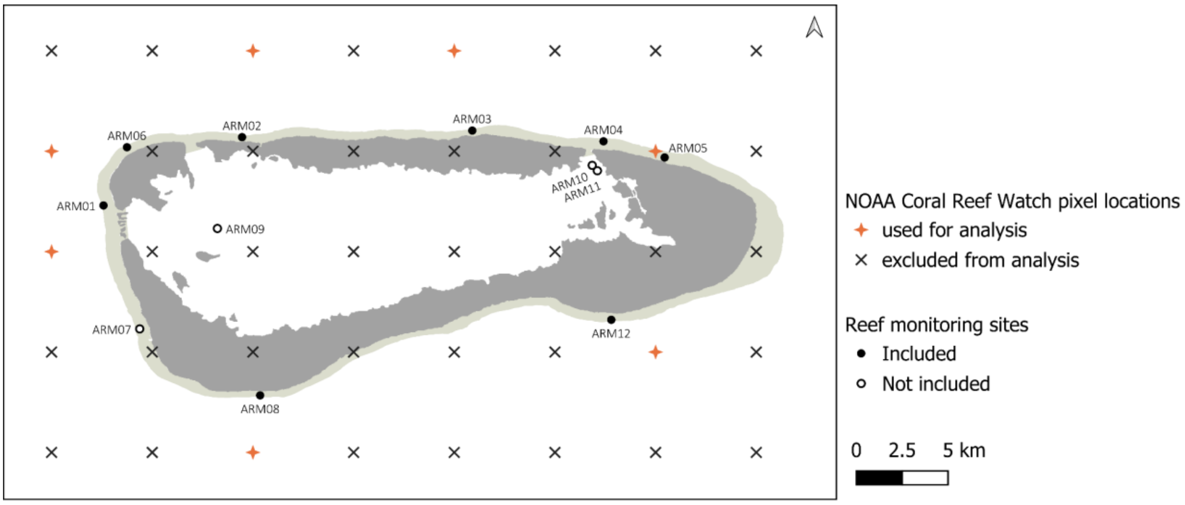

Figure S2: Aldabra map with the included survey sites and NOAA Coral Reef Watch raster cells overlaid. The top-left raster cell corresponds to -9.325, 46.175; the bottom right raster cell corresponds to -9.525, 46.525.

Table S2: *T*-test results, observed mean and standard error, and lower and upper 99% confidence limits from permutational analyses of the comparison of total and herbivorous fish biomass, coral juvenile density and structural complexity against reference thresholds. Results column reflects the original t-test result with changes in significance as a result of the permutational tests highlighted with **, either as prefix (Lower than threshold) or suffix (Higher than threshold).

Data	Site	t	df	*p*	Mean	SE	lower 99%CI	upper 99%CI	Result
Total fish biomass (kg ha^{-1})	Overall	3.3138	31	0.00235	1846.3	255.4	1242.1	4944.1	Higher than threshold
	ARM01	-0.08792	3	0.9355	986.2	157.3	696.8	1152.3	No difference
	ARM02	2.4607	3	0.0908	2382.6	561.9	1229.8	3107.8	No difference**
	ARM03	0.64463	3	0.5651	1282.2	437.8	735.4	2083.9	No difference
	ARM04	1.1173	3	0.3453	1709.2	634.8	1019.5	3047.3	No difference**
	ARM05	-0.02715	3	0.9800	994.1	216.5	626.7	1374.4	No difference
	ARM06	4.3192	3	0.0229	4267.1	756.4	3164.7	5289.2	Higher than threshold
	ARM08	0.9515	3	0.0660	1014.8	224.6	626.9	1399.7	No difference
	ARM12	1.2099	3	0.3130	2133.8	937.1	1457	4651.1	No difference**
Herbivore fish biomass (kg ha^{-1})	Overall	3.8482	31	0.0063	518.8	99.4	459.2	2221.2	Higher than threshold
	ARM01	1.516	3	0.2268	326.5	98.6	299	588.5	No difference**
	ARM02	1.712	3	0.1854	958.4	456.4	540.2	2104.7	No difference**
	ARM03	1.4076	3	0.2540	361.4	131.0	378.1	1153.9	No difference**
	ARM04	1.2097	3	0.3130	851.5	557.6	663	2257.5	No difference**
	ARM05	1.1194	3	0.3445	264.4	78.1	232.1	715.8	No difference**
	ARM06	1.4075	3	0.2540	500.0	229.5	279.4	1208.2	No difference**
	ARM08	1.3644	3	0.2658	446.5	197.6	348.5	891.6	No difference**
	ARM12	1.6439	3	0.1987	441.9	161.2	312	1019	No difference**
Coral juvenile abundance per m^2	Overall	5.5305	30	0.0000	14.2	1.5	9.6	27.1	Higher than threshold
	ARM01	4.0742	3	0.0267	17.2	2.7	14.2	21.8	Higher than threshold
	ARM02	3.8003	3	0.0320	25.8	5.2	13.9	27.9	Higher than threshold
	ARM03	1.1713	3	0.3260	9.2	2.6	7.6	14.6	No difference**
	ARM04	2.1075	2[a]	0.1696	12.5	3.0	8.9	15.4	No difference**
	ARM05	1.7892	3	0.1715	8.0	1.0	7.1	8.1	No difference**

Data	Site	t	df	*p*	Mean	SE	lower 99%CI	upper 99%CI	Result
	ARM06	3.6858	3	0.0346	18.2	3.3	12.9	22.9	Higher than threshold
	ARM08	2.7606	3	0.0701	15.8	3.5	9.7	20.4	No difference**
	ARM12	0.55641	3	0.6167	6.8	1.0	6.8	7.8	No difference**
Structural complexity	Overall	-0.13125	31	0.8964	3.1	0.2	1.5	4.5	No difference
	ARM01	4.5033	3	0.0205	3.8	0.1	3.6	3.9	Higher than threshold
	ARM02	8.2	3	0.0038	4.1	0.1	4.1	4.4	Higher than threshold
	ARM03	-0.4899	3	0.6578	3.0	0.2	2.6	3.4	No difference
	ARM04	1.3981	3	0.2565	3.9	0.6	2.8	4.8	No difference
	ARM05	-1.963	3	0.1444	2.3	0.4	1.1	2.9	**No difference
	ARM06	-0.28284	3	0.7957	3.0	0.4	2.6	3.9	No difference
	ARM08	-1.8	3	0.1697	2.9	0.1	2.6	2.9	**No difference
	ARM12	-9.3531	3	0.0026	1.8	0.1	1.6	1.9	Lower than threshold

[a] no data collected at 15 m depth

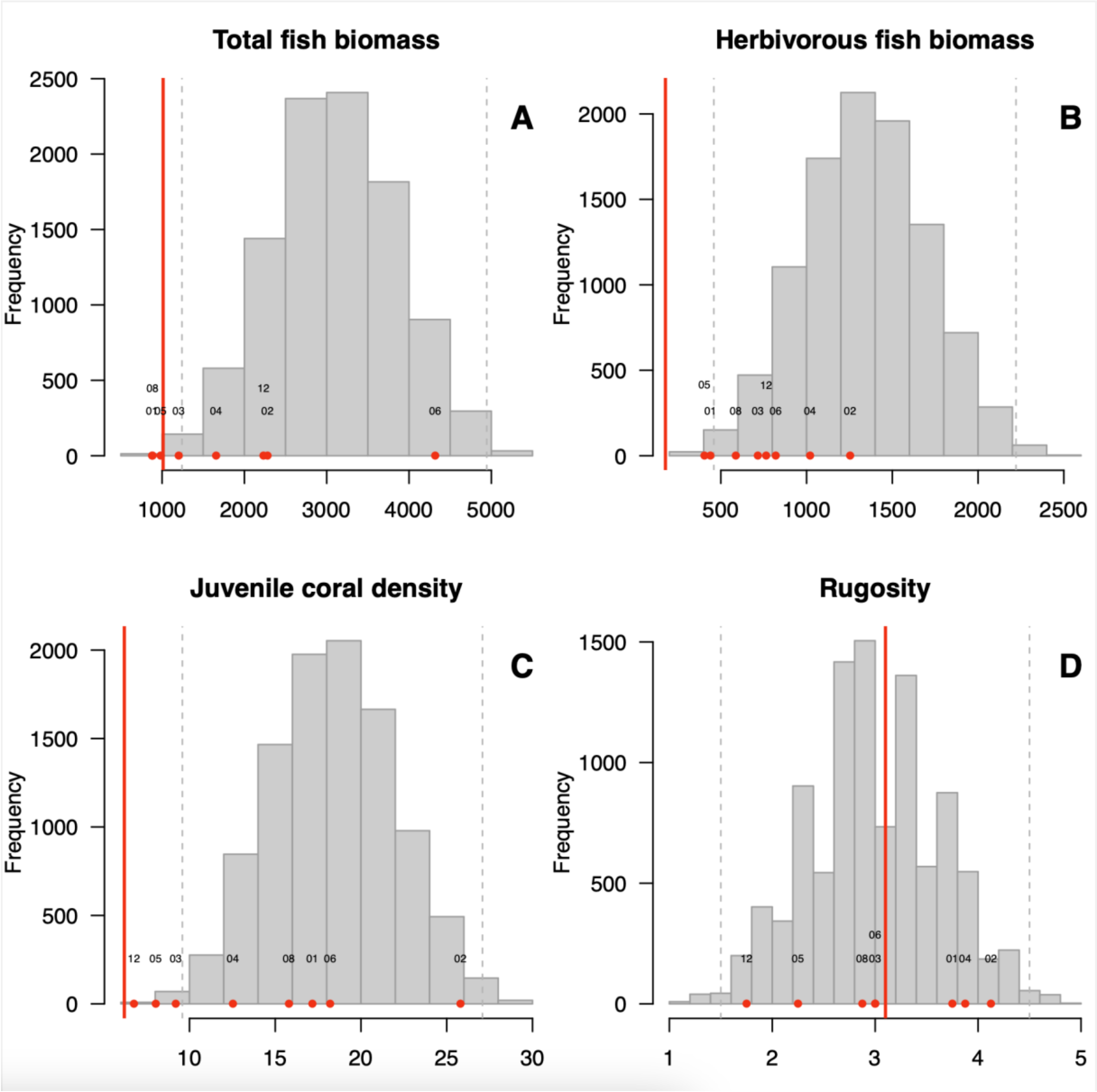

Figure S3: Atoll-level histograms of the pseudo-sampling from a sample population based on equal probability of pulling values (n=4; N=9999) from the range of transect-level values for each of the four reef characteristics (mean estimate). Units are A-B: kg per hectare, C: individuals per square metre, D: 6-point scale. Solid red line shows the theoretical threshold (A: MacNeil et al.[3]; B-D: Graham et al.[2]), red points show the observed means at each site, dashed grey lines show the 0.005 and 0.995 quantiles. We interpret a significant difference between the indicator and the recommended threshold when one or both quantiles do not overlap with the threshold value.

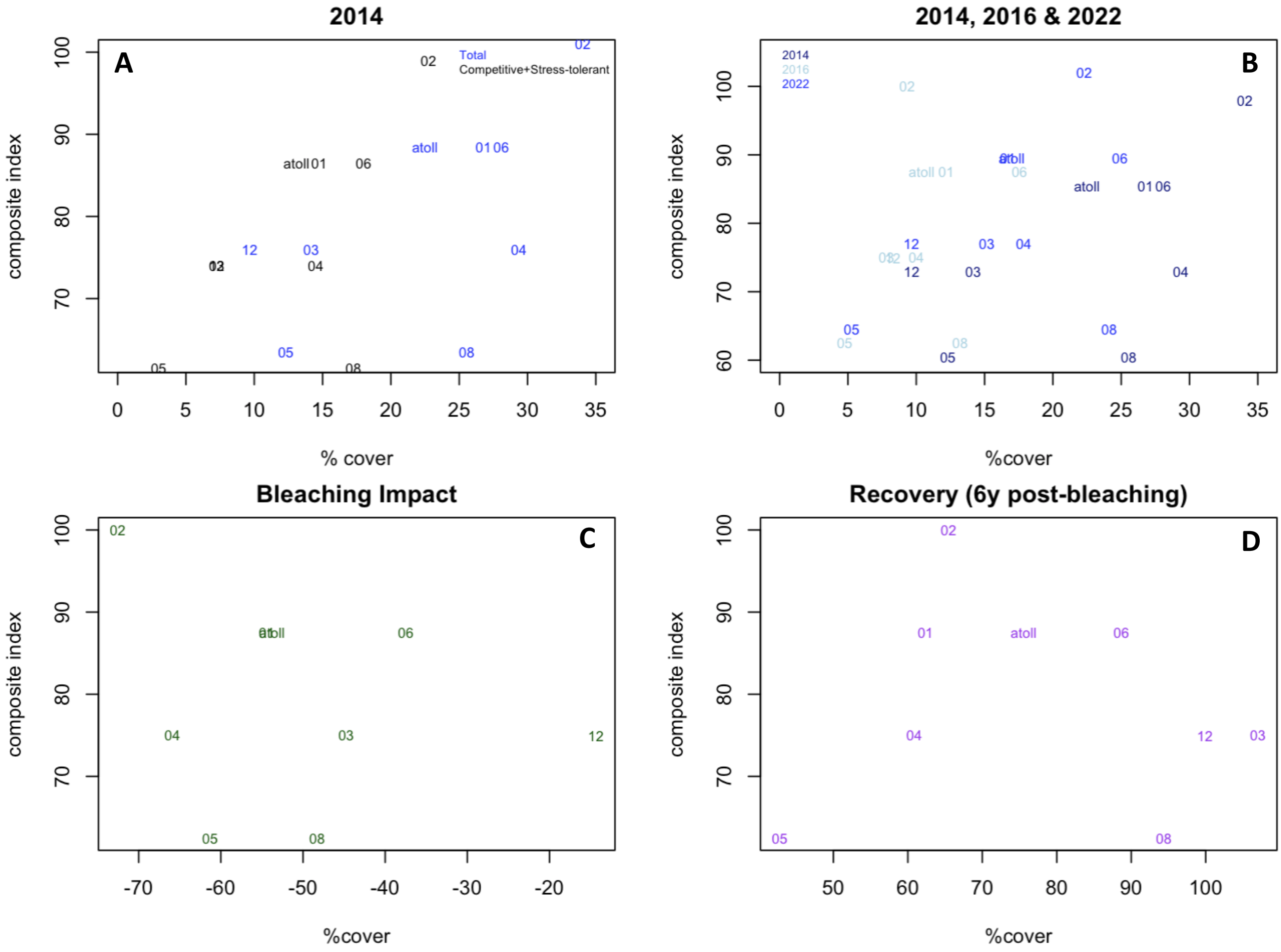

Figure S4: Composite index on the atoll level and for each site plotted against pre-bleaching percent coral cover: total and framework species (A), coral cover pre-and post-bleaching (B), percent reduction in coral cover post-bleaching (C) and percent recovery by 2020 (hard coral cover as percent of pre-bleaching hard coral cover; D).

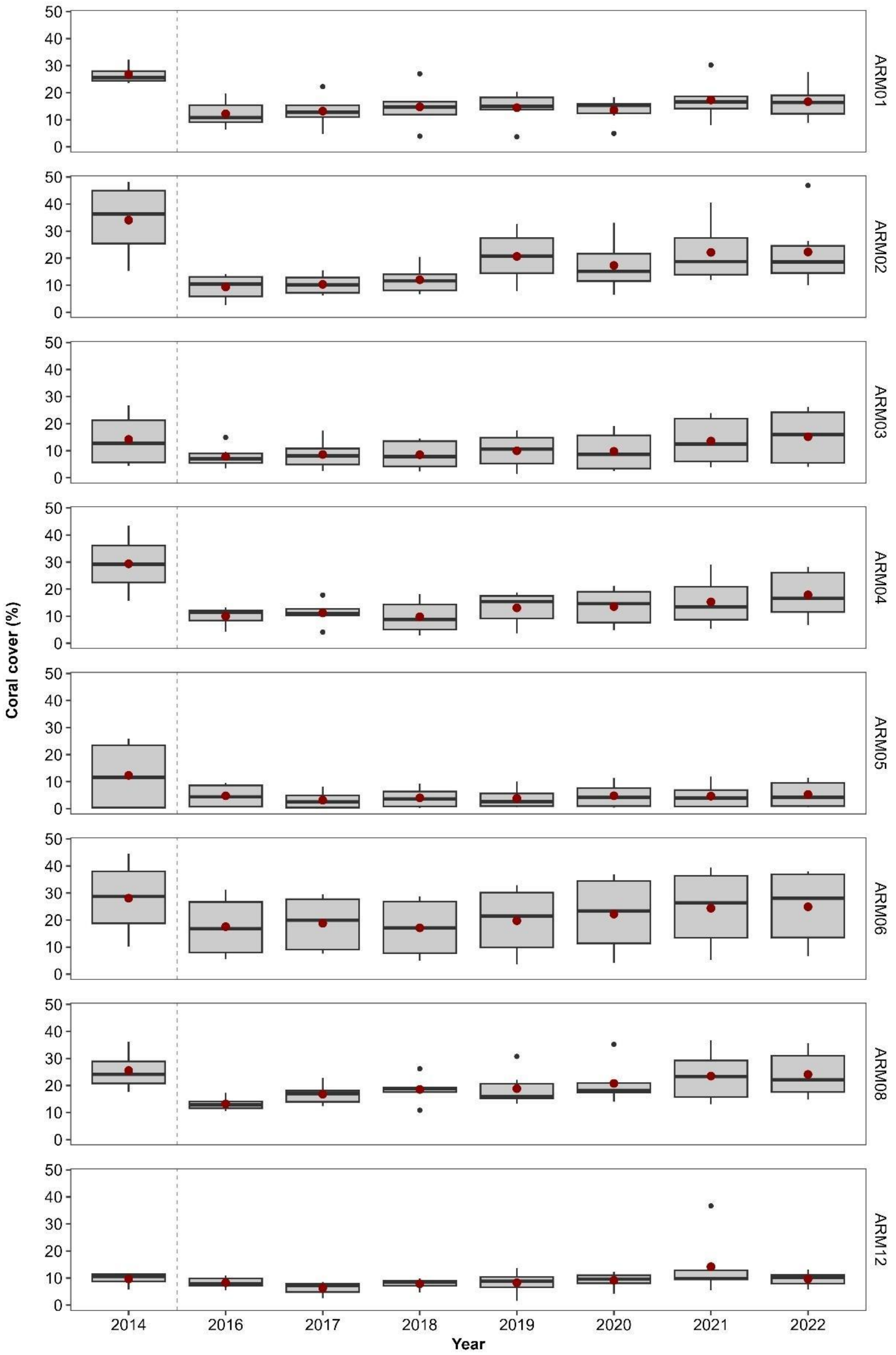

Figure S5: Percent coral cover across surveyed sites displayed as median and interquartile range (outliers: small black dots; means: larger red dots).

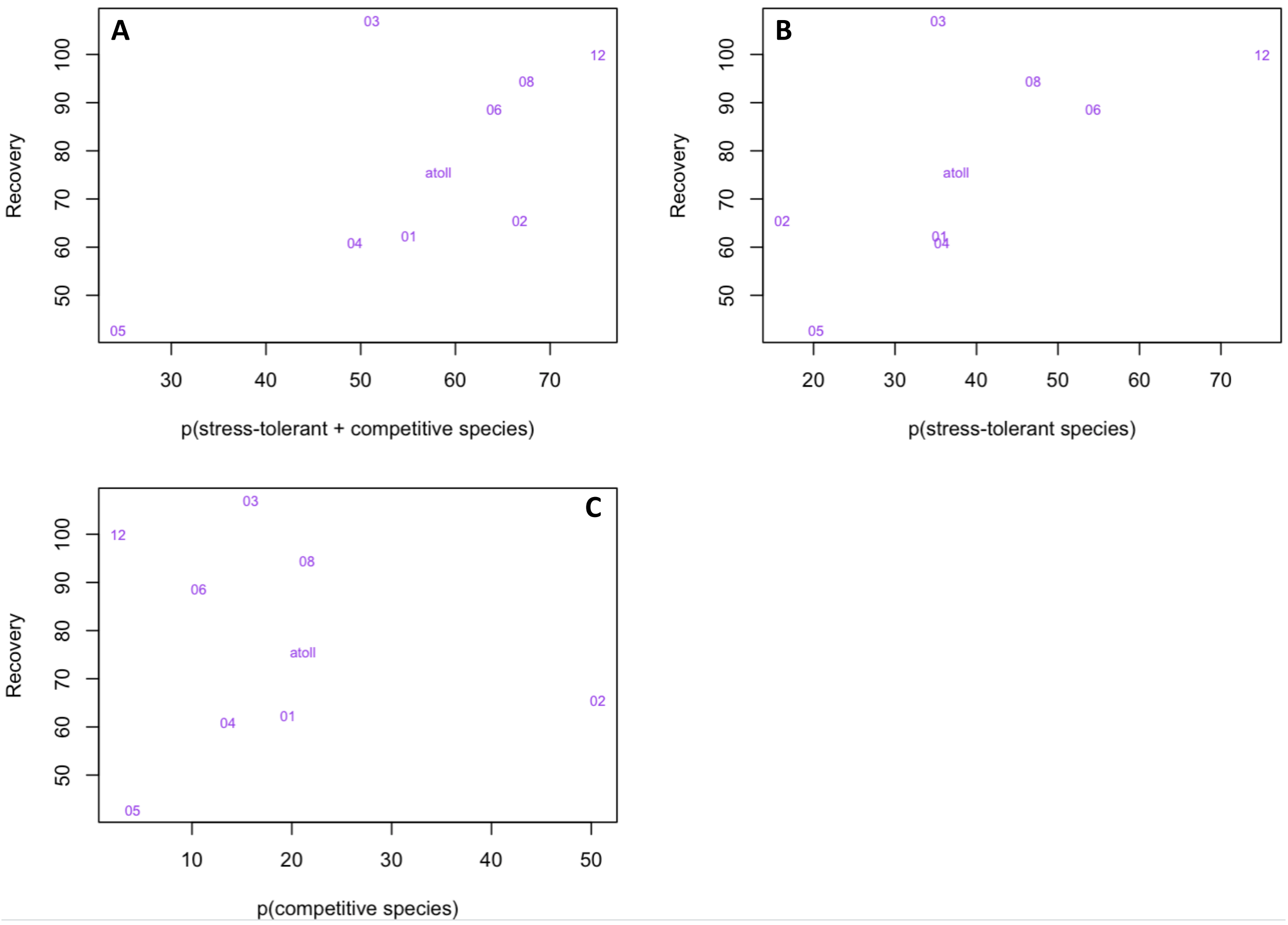

Figure S6: Recovery (hard coral cover in 2022 as percent of pre-bleaching hard coral cover) plotted against proportion of pre-bleaching hard coral cover made up of (A) framework coral species, (B) stress-tolerant coral species and (C) competitive coral species at the atoll level and for each site.

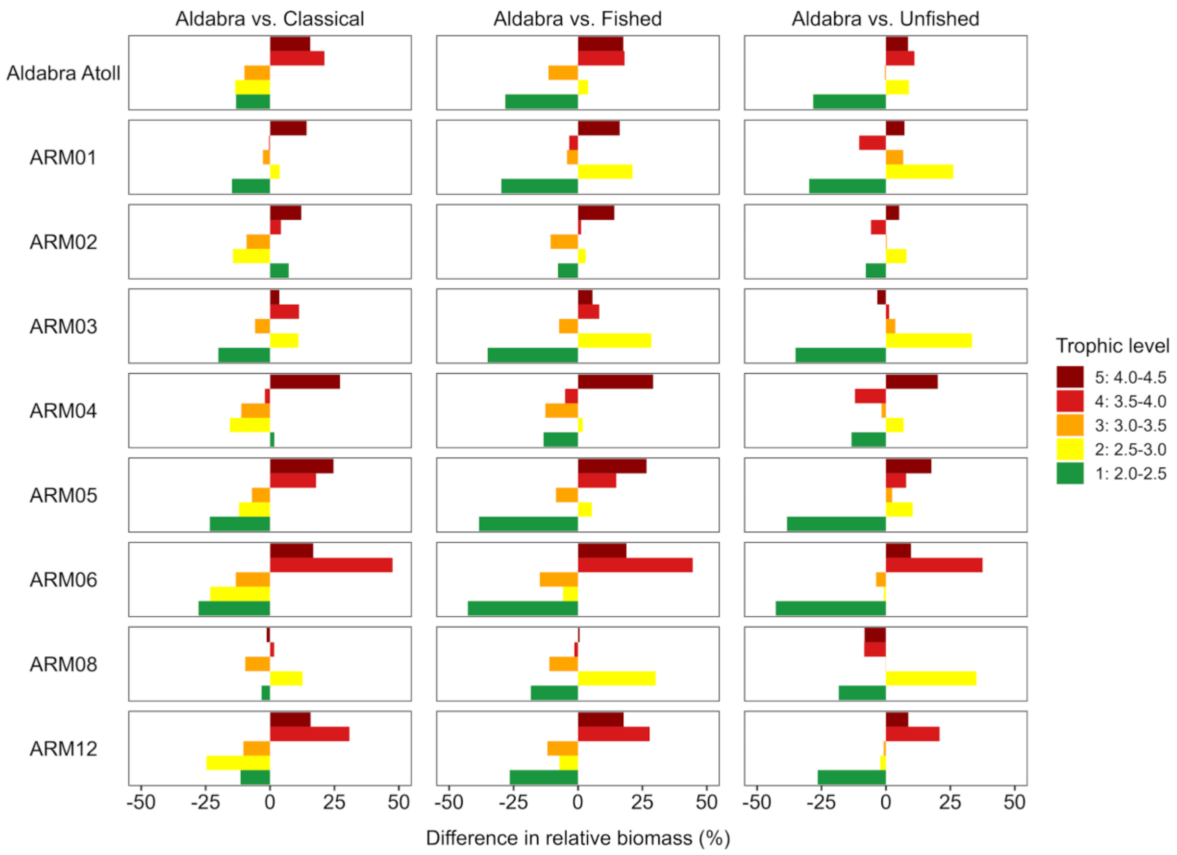

Figure S7: Difference in relative biomass per trophic level between Aldabra and the classic trophic pyramid (following Trebilco et al.[6]) and Aldabra and fished and unfished reefs (following Graham et al.[1]).

Table S3: Maximum degree heating weeks (obtained from NOAA Coral Reef Watch[7]) recorded close to each survey site.

Site	Date	Max. DHW	Distance raster cell to site (m)
ARM08	02/04/2016	5.41	3272
ARM01	07/04/2016	5.49	3839
ARM12	02/04/2016	5.63	2993
ARM06	07/04/2016	5.71	4121
ARM04	03/04/2016	5.88	2866
ARM05	03/04/2016	5.88	644
ARM02	03/04/2016	5.91	4854
ARM03	03/04/2016	5.95	4516

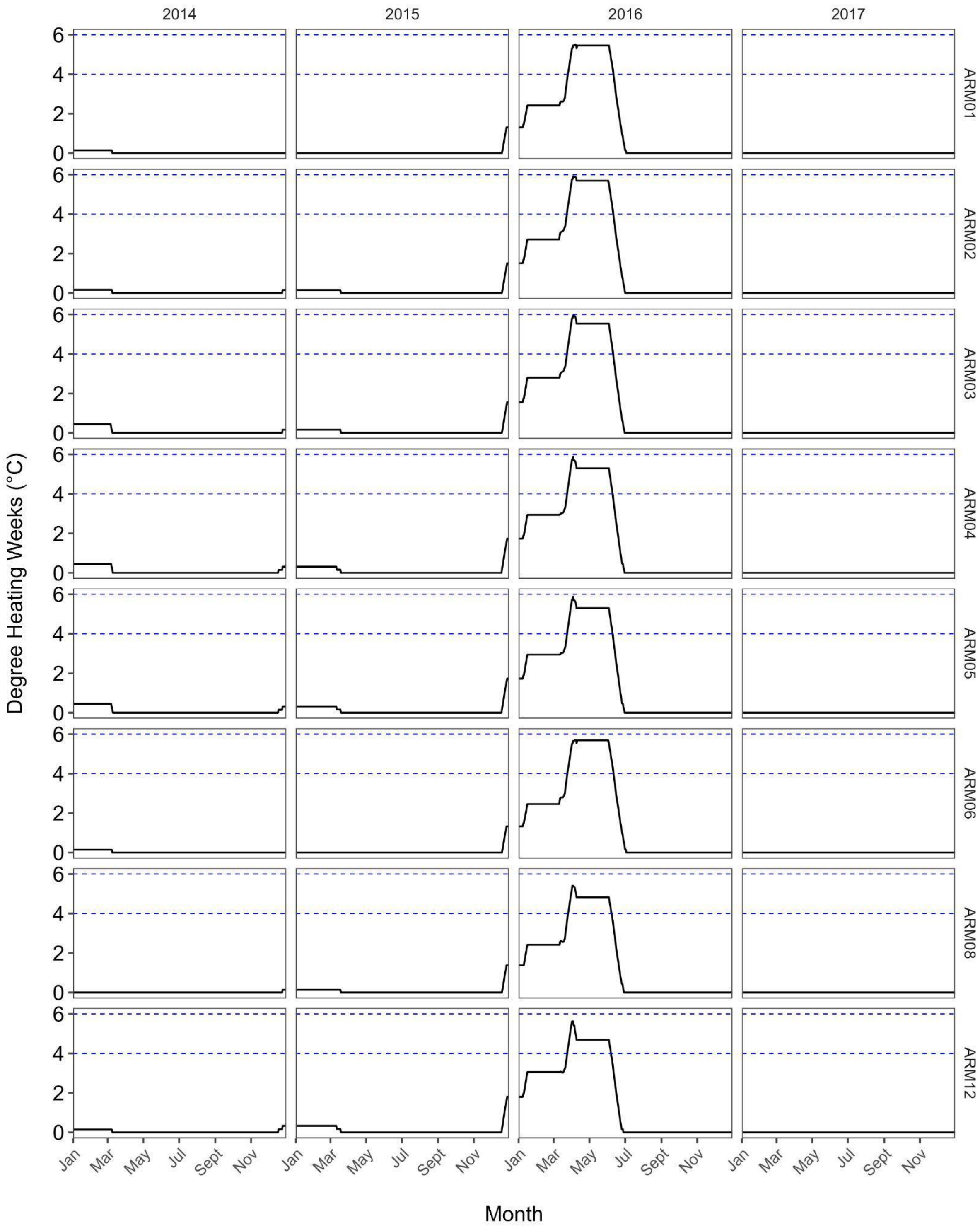

Figure S8: Degree Heating Weeks recorded at Aldabra between January 2014 and December 2017 (obtained from NOAA Coral Reef Watch[7]). For each survey site, data from the nearest raster cell was selected (see Figure S2 and Table S4). For ARM04 and ARM05 data from the same raster cell was used.

Table S4: *T*-test results, observed mean and standard error, and lower and upper 99% confidence limits from permutational analyses of the comparison of framework coral cover at Aldabra's sites against Darling et al.'s[8] 10% recommended threshold and the resulting management strategy. Results column reflects the original t-test result with changes in significance as a result of the permutational tests highlighted with **, either as prefix (Lower than threshold) or suffix (Higher than threshold).

Site	t	df	*p*	Mean	SE	lower 99%CI	upper 99%CI	Result	Management strategy	Proportion (%) of framework corals[a]
Overall	1.8186	31	0.07864	13.1	1.7	5.25	30.25	No difference**	Recover	58.2
ARM01	1.1613	3	0.3295	14.8	4.1	10	23	No difference	Recover	55.2
ARM02	1.8752	3	0.1574	22.8	6.8	8.8	31	No difference	Recover	66.9
ARM03	-0.8893	3	0.4394	7.3	3.1	4.5	13.8	No difference	Recover	51.4
ARM04	1.9335	3	0.1487	14.5	2.3	12	19	No difference**	Recover	49.3
ARM05	-3.9337	3	0.02962	3.0	1.8	1.5	5.5	Lower than threshold	Transform	24.4
ARM06	1.3303	3	0.2755	18.0	6.0	11	28	No difference**	Recover	64.1
ARM08	1.8578	3	0.1602	17.3	3.9	12.8	24.2	No difference**	Recover	67.8
ARM12	-1.457	3	0.2421	7.25	1.9	4.8	10.2	No difference	Recover	74.7

[a] relative to total hard coral cover

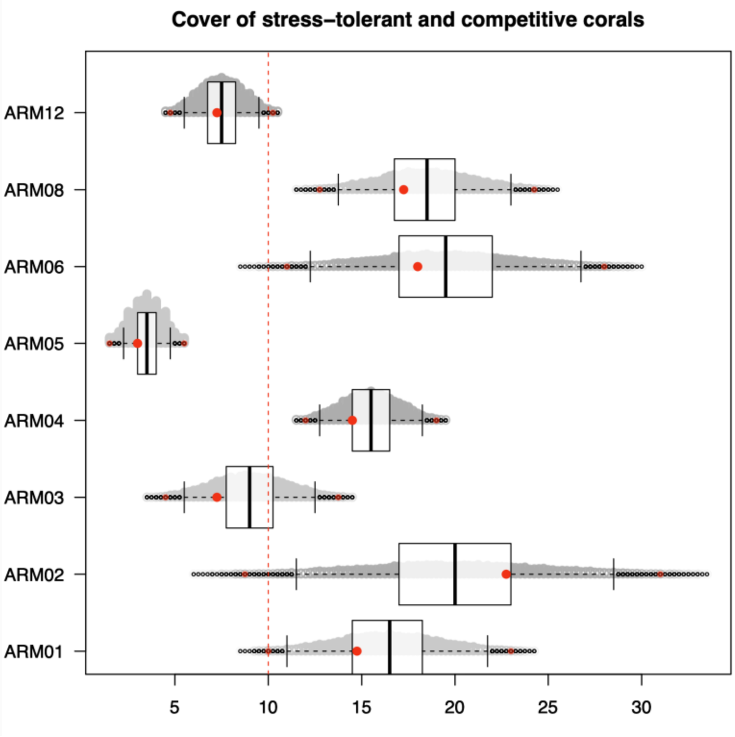

Figure S9: Distributions of the pseudo-sampling from site-level sample populations based on equal probability of pulling values (n=4 samples per pseudo-sample; N=9999 permutations) from the range of transect-level values for each of the four reef characteristics (mean estimate) overlaying stacked stripchart (pale grey circles) to show distribution of the pseudo-samples. Black vertical bars represent the 25th percentile, median, and 75th percentile from left to right. Whiskers extend to the largest value within 1.5 times the interquartile range (IQR) above the 75th percentile and the smallest value within 1.5 times the IQR below the 25th percentile. Dotted red line shows the theoretical threshold (10%; Darling et al.[8]), large red circles show the observed means, small red points show the 0.005 and 0.995 quantiles. We interpret a significant difference between the indicator and the recommended threshold when one or both quantiles do not overlap with the threshold value.

References

1. Graham, N. A. J. *et al.* Human disruption of coral reef trophic structure. *Current Biology* **27**, 231–236 (2017).
2. Graham, N. A. J., Jennings, S., MacNeil, M. A., Mouillot, D. & Wilson, S. K. Predicting climate-driven regime shifts versus rebound potential in coral reefs. *Nature* **518**, 94–97 (2015).
3. MacNeil, M. A. *et al.* Recovery potential of the world's coral reef fishes. *Nature* **520**, 341–344 (2015).
4. Guillou, N. Estimating wave energy flux from significant wave height and peak period. *Renewable Energy* **155**, 1383–1393 (2020).
5. Cheung, K. F. *WaveWatch III (WW3) Global Wave Model 2010, Updated 2021. Hourly 50 Km Satellite Time Series Data for Aldabra Atoll, Jan. 1, 2018–Jan. 1, 2025. Distributed by the Pacific Islands Ocean Observing System (PacIOOS). Http://Pacioos.Org/Metadata/Ww3_global.Html and Downloaded via the ERDAPP Data Server: Https://Pae-Paha.Pacioos.Hawaii.Edu/Erddap/Griddap/Ww3_global.Html. Accessed May 16 2025*.
6. Trebilco, R., Baum, J. K., Salomon, A. K. & Dulvy, N. K. Ecosystem ecology: size-based constraints on the pyramids of life. *Trends in Ecology & Evolution* **28**, 423–431 (2013).
7. NOAA Coral Reef Watch. *NOAA Coral Reef Watch Version 3.1 Daily 5 Km Satellite Time Series Data for Aldabra Atoll, Jan. 1, 2014–Dec. 31, 2017*. (College Park, Maryland, USA: NOAA Coral Reef Watch. Data accessed through Pacific Islands Ocean Observing System (PacIOOS) via ERDDAP, 2023).
8. Darling, E. S. *et al.* Social–environmental drivers inform strategic management of coral reefs in the Anthropocene. *Nat Ecol Evol* **3**, 1341–1350 (2019).
